# DISCOVER: A facile structure-based screening method for vinyl compound producing microbes

**DOI:** 10.1038/s41598-019-52518-6

**Published:** 2019-11-05

**Authors:** Yuji Aso, Mei Sano, Hikari Kuroda, Hitomi Ohara, Hiroshi Ando, Keiji Matsumoto

**Affiliations:** 1 Department of Biobased Materials Science, Kyoto Institute of Technology, 1 Hashigami-cho, Matsugasaki, Sakyo-ku, Kyoto 606-8585 Japan; 2 Corporate R&B Planning Department, Kaneka Corporation, 2-3-18 Nakanoshima, Kita-ku, Osaka 530-8288 Japan

**Keywords:** Soil microbiology, Applied microbiology

## Abstract

Here we report a novel structure-based microbial screening method for vinyl compound discovery, DISCOVER (direct screening method based on coupling reactions for vinyl compound producers). Through a two-step screening procedure based on selective coupling reactions of terminal alkenes, the thiol-ene reaction (1^st^ step of screening) and Mizoroki-Heck reaction, followed by iodine test (2^nd^ step of screening), microbes producing vinyl compounds like itaconic acid (IA) can be isolated from soil samples. In the 1^st^ step of screening, soil sources are plated on agar medium supplemented with an antimicrobial agent, α-thioglycerol (TG), and a radical initiator, VA-044 (VA). In the 2^nd^ step of screening, vinyl compounds produced in the cultures are labelled with iodobenzene via the Mizoroki-Heck reaction, followed by an iodine test, leading to the detection and characterisation of labelled products. We evaluated the validity of DISCOVER using IA and its producer *Aspergillus terreus*. Experimental data supported our hypothesis that IA reacts with TG in the medium via the thiol-ene reaction and consequently, *A. terreus* rapidly forms colonies on the agar medium because of the loss of the antimicrobial activity of TG. Using DISCOVER, high throughput and selective isolation of *A. terreus* strains producing IA was possible from soils.

## Introduction

To date, many screening methods for isolating microbes which produce value-added compounds have been developed, most of which are based on phenotypes (biological, chemical, and physiological properties) of the products^1^. For instance, researchers typically perform bioassays using indicator strains for screening of microbes which produce antibiotics, followed by structural analyses to identify them^2^. These conventional methods are based on phenotype screening. This approach is reasonable, even though desirable microbes could be missed, since the phenotype-based method is indirect and depends on the screening conditions, e.g. types of indicator strains used in bioassays. This bias is one of the biggest limitations to screening desired microbes.

Itaconic acid (IA) is a bio-vinyl compound produced by fungi such as *Aspergillus terreus*^3,4^, *Aspergillus itaconicus*^5^, *Ustilago maydis*^6^, *Candida* sp.^7^, *Rhodotorula* sp., and *Pseudozyma antarctica*^8^. IA is one of the most versatile and promising vinyl compounds since it possesses a terminal alkene exhibiting reactivity in radical polymerization. Because of its unique structure, IA is used worldwide for manufacturing materials such as plastics and bioactive compounds^9–11^. Therefore, isolation of a more suitable and high-titre IA producer from nature is desired. Fungi producing IA can be screened based on low culture pH. In fact, a novel IA producer, *P. antarctica*, has been isolated using a pH indicator, bromocresol purple^8^. This screening method is easy but does not distinguish between IA producers and other organic acid producers. In order to quickly and selectively isolate microbes producing IA on a large scale, structure-based screening in which the terminal alkene of IA can be utilized, is preferable.

**Figure 1:**
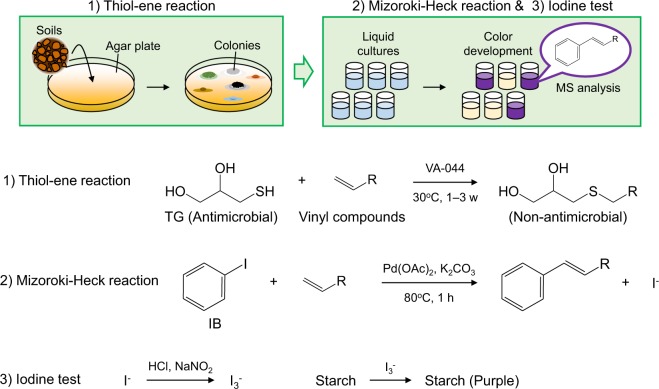
Overview of DISCOVER. Microbes producing vinyl compounds can be isolated from soil samples by the thiol-ene reaction (1^st^ step of screening) and the Mizoroki-Heck reaction followed by iodine test (2^nd^ step of screening). TG, α-thioglycerol; IB, iodobenzene.

The thiol-ene reaction^12^ and the Mirozoki-Heck reaction^13–16^ are two distinctive reactions of vinyl compounds with terminal alkenes. The thiol-ene reaction is a ‘click’ coupling reaction between a thiol group and an alkene (Fig. 1). The thiol group is radicalized by radical initiators, heat and/or light, resulting in the formation of a thiyl radical. Subsequently, the thiyl radical reacts with an alkene. The Mirozoki-Heck reaction is a coupling reaction between a haloalkane and an alkene that occurs in the presence of palladium catalysts under alkaline conditions. Recently, we developed a screening method for isolating IA producers from soil based on the Mirozoki-Heck reaction and followed by an iodine-test^17^. Using this method, we have isolated *A. terreus* which produces IA. To our knowledge, this is the first demonstration of a structure-based screening method that can be used to isolate microbes producing vinyl compounds. However, the screening efficiency is still low because the microbe cultures must be assayed individually using the Mizoroki-Heck reaction.

The aim of this study is to improve the screening efficiency of microbes producing vinyl compounds with a two-step screening method based on a selective cross-coupling reaction of terminal alkenes: the thiol-ene reaction (1^st^ step screening) and the Mizoroki-Heck reaction followed by an iodine test (2^nd^ step screening) (Fig. 1). We named this method DISCOVER (direct screening method based on coupling reaction for vinyl compound producers). In the 1^st^ step of screening, soil samples were spread on agar medium supplemented with an antimicrobial agent α-thioglycerol (TG) and a radical initiator 2,2′-azobis[2-(2-imidazolin-2-yl) propane] dihydrochloride (VA-044)^18^ (VA). It has been reported that TG shows antimicrobial activity against microbes such as *Escherichia coli*^19,20^ and *Aspergillus parasiticus*^21^. We hypothesized that vinyl compounds produced by microbes react with TG via thiol-ene reaction in the medium, resulting in loss of the antimicrobial activity of TG and subsequently, microbes producing vinyl compounds rapidly form colonies on the agar medium. In the 2^nd^ step of screening, vinyl compounds in the cultures were labelled with iodobenzene (IB) via the Mizoroki-Heck reaction and followed by an iodine-test, leading to detection and characterization of labelled products using liquid chromatography-mass spectrometry (LC-MS) analysis. The labelling reaction is dose- and reactivity-dependent. In this study, we optimized the conditions for the thiol-ene reaction to isolate microbes producing vinyl compounds after our hypothesis was proven. In conclusion, DISCOVER enabled us to screen favourable microbes producing vinyl compounds with high titre and/or reactivity.

## Results

### Characterisation of the thiol-ene reaction

**Figure 2:**
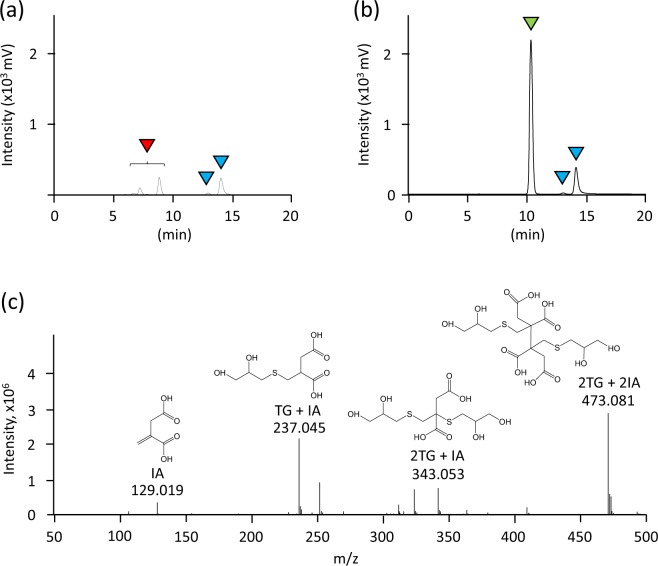
HPLC analysis of reactant of α-thioglycerol (TG) after the thiol-ene reaction with itaconic acid (IA) in the (**a**) presence or (**b**) absence of VA-044 (VA). 1.1 M TG and 0.55 M IA were mixed in water with or without 18 mM VA, followed by incubation at 80 °C for 1 day in a glass tube with a screw cap. The peaks labelled with blue, green, and red correspond to TG, IA, and the reactants of TG with IA, respectively. (**c**) MS analysis of reactants of TG after the thiol-ene reaction with IA in the presence of VA in negative ion mode.

**Figure 3:**
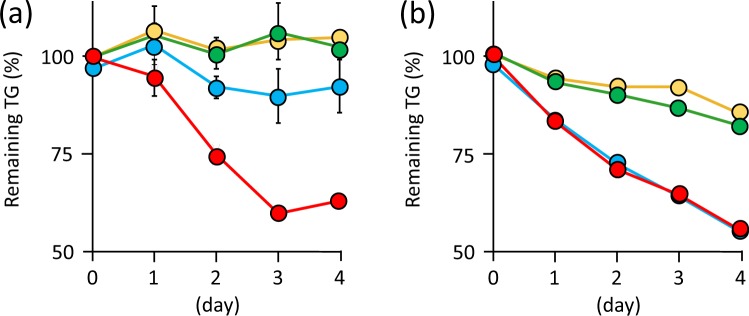
Reaction of α-thioglycerol (TG) with itaconic acid (IA) by the thiol-ene reaction under (**a**) anaerobic or (**b**) aerobic condition. 50 mM TG and/or 100 mM IA were mixed in water with or without 5 mM VA-044 (VA), followed by incubation at 30 °C for 4 days under anaerobic or aerobic condition. Yellow, 50 mM TG only; green, 50 mM TG + 100 mM IA; blue, 50 mM TG + 5 mM VA; red, 50 mM TG + 100 mM IA + 5 mM VA. After incubation, the concentration of the remaining TG in the reaction mixtures was analysed by the Ellman method^26^. This assay was performed at least in duplicate, and the average is represented with error bars indicating standard deviations.

Currently, no data is available on the thiol-ene reaction of TG with vinyl compounds produced by microbes in the presence of VA. To analyse the reaction, IA was used as a model compound in the thiol-ene reaction. Briefly, TG and IA were allowed to react in the presence of VA at 80 °C for 1 day and the peaks corresponding to the reactants of TG with IA were observed by high-performance liquid chromatography (HPLC) (Fig. 2a). TG exhibited two peaks corresponding to TG monomer (reduced form) and dimer (oxidized form) (Fig. 2a,b). Mass analysis depicted that TG reacted with IA in the following ratios 1:1 ([M-H]^−^ = 237.045 (observed); 237.043 (calculated)), 2:1 ([M-H]^−^ = 343.053 (observed); 343.052 (calculated)), and 2:2 ([M-H]^−^ = 473.081 (observed); 473.079 (calculated)) (Fig. 2c). These results indicated that the reaction of TG with IA indeed occurred in the presence of VA. To evaluate the effect of oxygen on the thiol-ene reaction, the reaction of TG with or without IA and VA was demonstrated in water under aerobic or anaerobic condition at 30 °C for 4 days. Under anaerobic conditions, the reaction rate was 37.5% when VA was added, while the reaction rate was almost zero when the radical initiator was omitted (Fig. 3a). The peaks corresponding to the reactants of TG with IA in the presence of VA were observed by HPLC analysis (data not shown). By contrast, the percentage of remaining TG in all cases was decreased under aerobic conditions (Fig. 3b). This result showed that aerobic conditions promote the formation of disulfide bond between TGs, thereby resulting in the consumption of TG. Similar results were obtained when the reactions were performed in potato dextrose (PD) broth (data not shown). Taken together, these results showed that the thiol-ene reaction of TG with IA occurs in the presence of VA and under anaerobic conditions.

**Figure 4:**
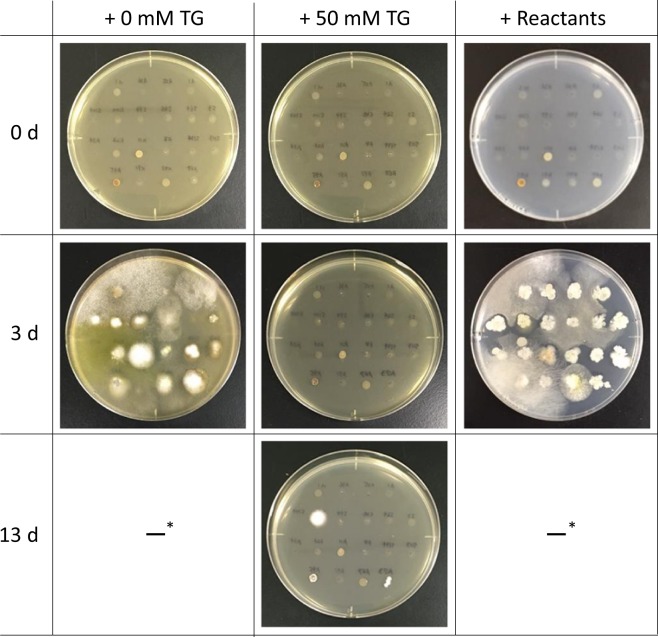
Microbial growth on the potato dextrose agar supplemented with 0 mM, 50 mM α-thioglycerol (TG), and the reactants of TG with itaconic acid (50 mM TG equivalent). After spotting 3 μL of the 20 soil supernatants onto the agar media, the agar media were incubated at 30 °C for 0, 3, and 13 days. *The photos of 13 days are not shown because the agar surfaces were entirely covered with the aerial mycelia.

Next, we hypothesized that vinyl compounds produced by microbes react with TG via the thiol-ene reaction in the medium, resulting in a loss of the antimicrobial activity of TG. To prove this, minimum inhibitory concentrations (MICs) of TG and its reactants with IA against 4 fungal and 2 bacterial strains (*A. terreus* NBRC 6123, *Aspergillus niger* NBRC 33023, *Aspergillus oryzae* NBRC 30113, *Saccharomyces cerevisiae* NBRC 10217, *Bacillus subtilis* subsp. *subtilis* NBRC 13179, and *E. coli* K-12) were determined using PD agar. In the case of TG, MICs were determined to be in the range of 12.5–100 mM (Table 1). These values were in accordance with those reported previously^19–21^. By contrast, MICs of the reactants of TG with IA were over 200 mM in all cases, suggesting a loss of the antimicrobial activity of TG. These results indicated that the thiol-ene reaction of TG with IA leads to a loss of the antimicrobial activity of TG. A similar result was obtained when the antibacterial activity of the reactants of TG with IA against soil microbes was tested (Fig. 4), supporting our hypothesis.

**Table 1:** Minimum inhibitory concentrations (MICs) of α-thioglycerol (TG) and its reactants with itaconic acid (IA) against fungal and bacterial strains.

Strains	MICs	
	TG	^a^Reactants
*Aspergillus terreus* NBRC 6123	12.5	>200
*Aspergillus niger* NBRC 33023	50	>200
*Aspergillus oryzae* NBRC 30113	25	>200
*Saccharomyces cerevisiae* NBRC 10217	50	>200
*Bacillus subtilis* subsp. *subtilis* NBRC 13179	100	>200
*Escherichia coli* K-12	25	>200

^a^The reactants of TG with IA (TG equivalent).

**Figure 5:**
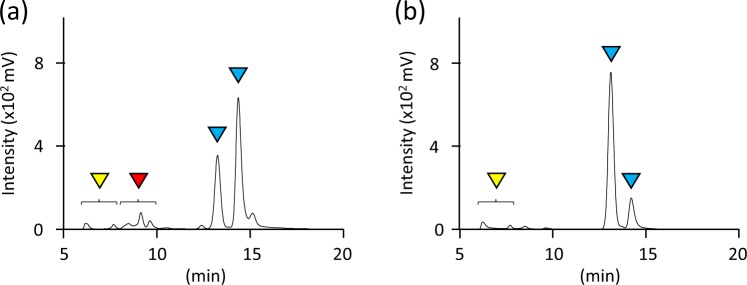
(**a**) HPLC analysis of the culture supernatant of *A. terreus* NBRC 6123 in the potato dextrose (PD) broth liquid medium with 50 mM α-thioglycerol (TG) and 0.5 mM VA-044 (VA). (**b**) PD broth liquid medium with 50 mM TG and 0.5 mM VA was analysed as a control. Each medium was incubated for 4 weeks at 30 °C. The peaks labelled with blue, red, and yellow correspond to TG, the reactants of TG with itaconic acid, and components of PD broth liquid medium, respectively.

For screening microbes using the thiol-ene reaction, the optimum concentrations of TG and VA were determined by colony formation of an IA producer *A. terreus* NBRC 6123 on PD agar containing TG and VA at various concentrations. *A. terreus* NBRC 6123 displayed no growth on agar containing 50 mM TG without VA while the strain formed a colony when 0.5 mM VA was included (data not shown). Contrastingly, *A. terreus* NBRC 6123 showed no growth on agar containing more than 60 mM TG even in the presence of 0.5 mM VA (data not shown). When *A. terreus* NBRC 6123 was cultivated in PD liquid medium with 50 mM TG and 0.5 mM VA, the reactants of TG with IA were observed in the culture supernatant (Fig. 5). This implies that IA produced by *A. terreus* NBRC 6123 reacted with TG in the medium during cultivation. These results denote that the optimum concentrations of TG and VA were 50 mM and 0.5 mM, respectively, for screening IA producers.

### Evaluation of the screening agar medium

The selectivity of PD agar containing 50 mM TG and 0.5 mM VA was evaluated for three *Aspergillus* fungal strains, *A. terreus* NBRC 6123, *A. niger* NBRC 33023, and *A. oryzae* NBRC 30113. *A. terreus* NBRC 6123 formed colonies after 5 days but the others formed colonies after 9 days (data not shown). MICs of TG against the former and the latter strains were 12.5 mM and 25–50 mM, respectively (Table 1). These results evidently suggest that the thiol-ene reaction promotes colony formation of microbes producing vinyl compounds on the agar medium. To characterise the selective function of the agar medium, re-isolation of *A. terreus* NBRC 6123 from soil samples was demonstrated using two different agar media, and then the appearance and isolation rates were calculated. Appearance rates were 104% and 108% when PD and GM1 agars were used, respectively (Table 2). This finding indicated that the media ingredients did not affect the number of colonies produced by *A. terreus* NBRC 6123. Isolation rates were 33% and 96% in the case of PD agar medium with and without TG and VA, respectively. Interestingly, although most microbes found in the soil rarely form colonies in the case of PD agar medium with TG and VA, *A. terreus* NBRC 6123 did. A similar result was obtained with the GM1 agar medium. Isolation rates were 60% and 100% with and without TG and VA, respectively, indicating that the screening method based on the thiol-ene reaction is not dependent on the type of agar medium.

**Table 2:** Re-isolation of *Aspergillus terreus* NBRC 6123 from soil samples using potato dextrose (PD) agar and GM1 agar with or without 50 mM α-thioglycerol (TG) and 0.5 mM VA-044 (VA).

Agar media	Plating of spores of *A. terreus*		Plating of spores of *A. terreus* together with soil samples		
	Number of colonies of *A. terreus*	Appearance rate (%)	Number of colonies of *A. terreus*	Number of colonies of other soil microbes	Isolation rate (%)
PD, no ingredients	22 ± 0^*^	104	20 ± 1	40 ± 0	33^**^
PD, +50 mM TG, 0.5 mM VA	23 ± 8^*^		27 ± 13	1 ± 0	96^**^
GM1, no ingredients	23 ± 6^*^	108	17 ± 0	11 ± 0	60^***^
GM1, +50 mM TG, 0.5 mM VA	25 ± 1^*^		20 ± 2	0	100^***^

^*^No significant difference among the number of colonies (*P* > 0.05); ^**^significant difference between the isolation rates (*P* < 0.01);

^***^significant difference between the isolation rates (*P* < 0.05).

Collectively, the findings demonstrate that agar medium containing 50 mM TG and 0.5 mM VA is the most suitable medium to screen microbes producing vinyl compounds based on the thiol-ene reaction. In combination with another screening method based on the Mizoroki-Heck reaction, which we have reported previously^17^, a novel microbial screening method, DISCOVER, was developed.

### Screening of microbes producing vinyl compounds from soil samples by DISCOVER

**Figure 6:**
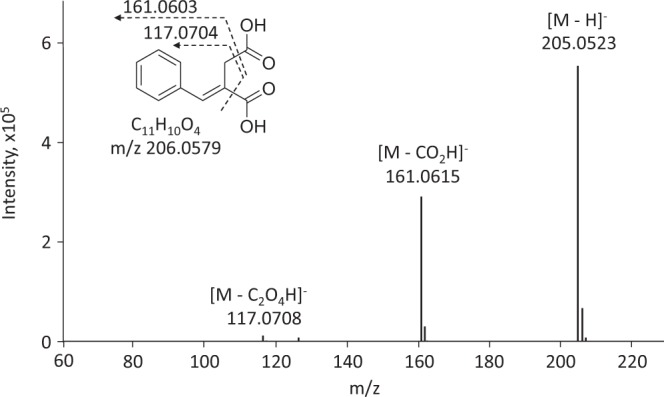
Mass analysis of the culture of S13-1 after labelling reaction by the Mizoroki-Heck reaction.

We attempted to screen microbes producing vinyl compounds from soil samples by DISCOVER. In the 1^st^ step of screening, 98 soil samples were plated onto PD and GM1 agar media containing 50 mM TG and 0.5 mM VA, resulting in 85 colonies. For cultivation in liquid medium, GM2, which contains glycerol instead of glucose as the carbon source, was used in order to avoid the appearance of pseudo-strains during the iodine-test when glucose was utilized (data not shown). In the 2^nd^ step of screening, the Mizoroki-Heck reaction was demonstrated with the cultures and subsequently, the iodine-test was performed, resulting in 45 strains that tested iodine positive. The reactants of the Mizoroki-Heck reaction were detected between 12–13 min on the HPLC chromatograms when IB was added (Fig. S1). IA labelled with IB was detected at 12.5 min on the chromatogram, suggesting that most of the 45 strains produce IA. One isolate, namely S13-1, was used for further investigation. Sequence analysis of the 26S rRNA gene identified the S13-1 strain as *A. terreus*. Further, mass analysis validated the monoisotopic mass and molecular formula of the labelled product by the Mizoroki-Heck reaction as 205.0523 [M-H]^−^ and C_11_H_10_O_4_, respectively (Fig. 6). Additionally, fragment ions (observed at, 161.0615 [M-CO_2_H]^−^ and 117.0708 [M-C_2_O_4_H]^−^; with calculated molecular weight 161.0603 [M-CO_2_H]^−^ and 117.0704 [M-C_2_O_4_H]^−^) corresponding to ionized decarboxylated-products were observed. These products correspond to those of 2-benzylidenesuccinic acid which is the reactant of IA with IB in the Mizoroki-Heck reaction. From these observations, we concluded that the S13-1 strain is identical to the IA producer *A. terreus*.

## Discussion

In this study, a facile structure-based microbial screening method for vinyl compound discovery, DISCOVER, was developed (Fig. 1). This method is based on two distinctive reactions of vinyl compounds with terminal alkenes, the thiol-ene reaction and the Mirozoki-Heck reaction. TG is a key compound in the thiol-ene reaction step. TG is known to interfere with various physiological processes in cells^19–25^. This is the first report on screening of microbes using the thiol-ene reaction. We proved the principle of this method and subsequently determined optimal conditions of the thiol-ene reaction.

We performed the thiol-ene reaction at 30 °C, unless otherwise specified, since this temperature is thought to be preferable for the cultivation of most soil microbes. The thiol-ene reaction was performed using VA because the radical initiator is water-soluble and has a 10-hour half-life decomposition temperature of 44 °C. We performed the thiol-ene reaction at 80 °C (Fig. 2) so that the reaction was complete after 1 day because the reaction was completed at 30 °C after several days (data not shown). Several complexes were observed after the reaction of TG with IA by the thiol-ene reaction (Fig. 2), arising from a radical–radical coupling process (termination reaction)^12^. Therefore, we used the mixture of the products as the reactants when the MICs of the reactants were evaluated. VA is necessary (Fig. 3) for the progress of the thiol-ene reaction and an oxidizing atmosphere results in the oxidization of TG. Our screening method is performed on an agar medium, and the inside of the agar medium is thought to be anaerobic; thus, suggesting that the reaction of TG with vinyl compounds produced by microbes occurs on the inside rather than on the surface of the agar medium.

MIC tests showed the antimicrobial activity of TG against fungi and bacteria seemed to be diminished after the thiol group of TG was coupled with IA (Table 1). This indicates that the thiol group of TG is involved in the antimicrobial activity (Fig. 4 and Table 2). Most microbes in soil cannot form colonies on agar medium supplemented with TG even in the presence of VA. Interestingly, an IA producer *A. terreus* NBRC 6123 showed the lowest MIC among microbes tested (Table 1). Along with other results (Table 2), this indicates that an IA producer is sensitive to TG but it can form colonies rapidly compared to other microbes via the thiol-ene reaction. The thiol-ene reaction of TG with IA (Fig. 5) produced by *A. terreus* NBRC 6123 occurred in the medium and not on the surface; in other words, it can be called an “*in situ* thiol-ene reaction”.

Using DISCOVER, we attempted to screen microbes producing vinyl compounds from 98 soil samples. As a result, isolation efficiency of iodine-test positive strains from soil samples was calculated at 53%. The efficiency was only 15% with 2^nd^ step screening only (data not shown). This shows that the 1^st^ step of screening based on the thiol-ene reaction enriches iodine-test positive strains. An isolated strain, S13-1, was identified as *A. terreus* and mass analysis showed that this strain produces IA. HPLC analysis revealed that most of the 45 strains isolated after iodine-test produced IA, but interestingly, a few of them showed unique peaks unrelated to IA (Fig. S1). This suggests that these strains produced novel vinyl compounds.

In conclusion, a novel screening method, DISCOVER, enables large scale testing and it selectively screens microbes that produce vinyl compounds with terminal alkenes. To our knowledge, this is the first report on a structure-based method for isolating microbes producing vinyl compounds with terminal alkenes. DISCOVER could pave the way to isolate novel microbes producing vinyl compounds acting as biomonomers with radical polymerization activities.

## Materials and Methods

### Bacterial strains, media, and soil samples

*A. terreus* NBRC 6123, *A. niger* NBRC 33023, *A. oryzae* NBRC 30113, *S. cerevisiae* NBRC 10217, and *B. subtilis* subsp. *subtilis* NBRC 13179 were obtained from the National Institute of Technology and Evaluation (Chiba, Japan). *E. coli* K-12 (ATCC 10798) was obtained from the American Type Culture Collection (VA, USA).

Microbes were grown in Difco PD broth (Becton, Dickinson and Company, MD, USA), GM1 medium (for 1 L: 130 g glucose, 0.1 g KH_2_PO_4_, 3 g NH_4_NO_3_, 1 g MgSO_4_·7H_2_O, 3.75 g CaCl_2_, 1.25 mg FeCl_2_·4H_2_O, 8 mg ZnSO_4_·7H_2_O, 15 mg CuSO_4_·5H_2_O) or GM2 medium (for 1 L: 130 g glycerol, 0.1 g KH_2_PO_4_, 3 g NH_4_NO_3_, 1 g MgSO_4_·7H_2_O, 3.75 g CaCl_2_, 1.25 mg FeCl_2_·4H_2_O, 8 mg ZnSO_4_·7H_2_O, 15 mg CuSO_4_·5H_2_O) at 30 °C. When the solid media were required, 15 g/L agar was added to the media.

Ninety-eight soil samples were obtained from several places in Japan. About 1 g of each soil sample was resuspended in 1 mL of sterilized water and subsequently gently mixed. After allowing the samples to settle for 10 min, 3 μL of the supernatant was spotted onto PD agar.

### Characterization of the thiol-ene reaction

To confirm the reaction of an antimicrobial agent TG (Tokyo Chemical Industry Co., Ltd., Tokyo, Japan) with IA in the presence of a radical initiator VA (Wako Pure Chemical Industries, Ltd., Osaka, Japan), 1.1 M TG, 0.55 M IA, and 18 mM VA were mixed with water, followed by incubation at 80 °C for 1 day in a glass tube with a screw cap. After incubation, the reactants were characterized using mass spectrometry and based on MICs. Prior to the experiment on MICs, remaining TG in the reaction mixture was eliminated using Sep-Pak tC18 Plus Light Cartridge (Waters, MA, USA) with water and acetonitrile as eluants. The reactants were resuspended with water and subsequently pH of the reactant solution was adjusted to 5.0 with ammonium solution because the pH of the TG solution is about 5.0.

The reactants of TG with IA were quantified using a Prominence HPLC system (Shimadzu, Kyoto, Japan) equipped with an ion-exclusion column, Shim-pack SCR-102H (Shimadzu) and an UV detector. The reactants were eluted using a 0.1% perchloric acid solution with a flow rate of 0.9 mL/min. Absorbance of the eluate was monitored at 210 nm. Mass determination of the reactants was performed by a Prominence HPLC system (Shimadzu) equipped with a reverse phase column, Cadenza CD-C18 (Imtakt, Kyoto, Japan), coupled with mass spectrometry micrOTOF II (Bruker, MA, USA) as a detector. The reactants were eluted using a 0.1% formic acid/acetonitrile solution with a flow rate of 0.2 mL/min. Absorbance of the eluate was monitored at 210 nm and mass analysis was operated in negative ion mode.

To investigate the effects of oxygen and VA on the thiol-ene reaction, 50 mM TG and 100 mM IA were mixed in water with or without 5 mM VA, followed by incubation at 30 °C for 4 days under anaerobic or aerobic condition. After incubation, the concentration of remaining TG in the reaction mixtures was analysed by the Ellman method^26^. Briefly, 100 μL of the reaction mixture after the thiol-ene reaction, 50 μL of 10 mM Ellman reagent (5,5′-Dithiobis(2-nitrobenzoic acid) (Tokyo chemical industry Co. Ltd., Tokyo, Japan)), and 50 μL of 500 mM Tris-HCl (pH 8.0) were mixed and subsequently incubated at 30 °C for 1 h in a closed microtube. Absorbance of the mixture was measured at 412 nm and then the concentration of TG was calculated using a standard curve.

### Measurement of MICs

Microbial growths were evaluated on PD agar supplemented with 50 mM TG or the reactants of TG with IA (50 mM TG equivalent) by spotting 3 μL of the 20 soil supernatants onto the agar medium, followed by incubation at 30 °C for 13 days.

After cultivation of the strains *A. terreus* NBRC 6123, *A. niger* NBRC 33023, and *A. oryzae* NBRC 30113, the spores and aerial mycelia were scraped off the agar surface with a scraper. The suspensions were passed through a glass filter with 0.8% NaCl solution containing 0.1% Tween 80 to remove the mycelia, and the collected spores were resuspended in the solution. The number of spores in the solutions was counted using a haemocytometer. The cultures of *S. cerevisiae* NBRC 10217, *B. subtilis* subsp. *subtilis* NBRC 13179, and *E. coli* K-12 were plated onto PD agar and then incubated at 30 °C. The number of viable cells in the cultures was determined by the colony count method.

Five to twenty spores or viable cells were spotted onto PD agar with 0–200 mM TG or the reactants of TG with IA (0–200 mM TG equivalent). After incubation at 30 °C for 3 days until colonies appeared., cell growth on the agar medium was evaluated.

### Evaluation of the screening agar medium

To determine the optimum concentrations of TG and VA, PD agar medium supplemented with 0–80 mM TG and 0–5 mM VA were prepared. The ingredients were added to the agar media without being autoclaved. Ten viable spores of *A. terreus* NBRC 6123 were plated onto the agar plates, followed by incubation at 30 °C for 10 days until colonies appeared.

The growth rates of *A. terreus* NBRC 6123, *A. niger* NBRC 33023, and *A. oryzae* NBRC 30113 on PD agar supplemented with 50 mM TG and 0.5 mM VA were investigated. One hundred viable spores of these strains were plated onto the agar medium and then incubated at 30 °C for 9 days until colonies appeared. Colony forming time was defined as the approximate time it took to form a colony 1 mm in diameter.

Re-isolation of *A. terreus* from soil samples was demonstrated. Fifteen to thirty viable spores of *A. terreus* NBRC 6123 were added to 1 mL of supernatant of soil mixture consisting of 20 soil samples. The spores of *A. terreus* with or without the supernatant of the soil mixture were plated onto PD agar or GM1 agar with 50 mM TG and 0.5 mM VA. After incubation at 30 °C for 8 days until colonies appeared, the number of colonies of *A. terreus* and other soil microbes on the agar media was counted. A colony of *A. terreus* is distinguishable from those of other soil microbes because of its orange colour. Appearance and isolation rates were defined as follows.

$$\begin{array}{c}{\rm{A}}{\rm{p}}{\rm{p}}{\rm{e}}{\rm{a}}{\rm{r}}{\rm{a}}{\rm{n}}{\rm{c}}{\rm{e}}\,{\rm{r}}{\rm{a}}{\rm{t}}{\rm{e}}({\rm{ \% }})={\rm{n}}{\rm{u}}{\rm{m}}{\rm{b}}{\rm{e}}{\rm{r}}\,{\rm{o}}{\rm{f}}\,{\rm{c}}{\rm{o}}{\rm{l}}{\rm{o}}{\rm{n}}{\rm{i}}{\rm{e}}{\rm{s}}\,{\rm{o}}{\rm{f}}\,A.\,terreus\,{\rm{o}}{\rm{n}}\,{\rm{t}}{\rm{h}}{\rm{e}}\,{\rm{a}}{\rm{g}}{\rm{a}}{\rm{r}}\,{\rm{m}}{\rm{e}}{\rm{d}}{\rm{i}}{\rm{a}}\,{\rm{w}}{\rm{i}}{\rm{t}}{\rm{h}}\,50\,{\rm{m}}{\rm{M}}\,{\rm{T}}{\rm{G}}\,{\rm{a}}{\rm{n}}{\rm{d}}\,0.5\,{\rm{m}}{\rm{M}}\,{\rm{V}}{\rm{A}}/{\rm{n}}{\rm{u}}{\rm{m}}{\rm{b}}{\rm{e}}{\rm{r}}\,{\rm{o}}{\rm{f}}\,{\rm{c}}{\rm{o}}{\rm{l}}{\rm{o}}{\rm{n}}{\rm{i}}{\rm{e}}{\rm{s}}\,{\rm{o}}{\rm{f}}\,A.\,terreus\,{\rm{o}}{\rm{n}}\,{\rm{t}}{\rm{h}}{\rm{e}}\,{\rm{a}}{\rm{g}}{\rm{a}}{\rm{r}}\,{\rm{m}}{\rm{e}}{\rm{d}}{\rm{i}}{\rm{a}}\,{\rm{w}}{\rm{i}}{\rm{t}}{\rm{h}}{\rm{o}}{\rm{u}}{\rm{t}}\,{\rm{T}}{\rm{G}}\,{\rm{a}}{\rm{n}}{\rm{d}}\,{\rm{V}}{\rm{A}}.\\ {\rm{I}}{\rm{s}}{\rm{o}}{\rm{l}}{\rm{a}}{\rm{t}}{\rm{i}}{\rm{o}}{\rm{n}}\,{\rm{r}}{\rm{a}}{\rm{t}}{\rm{e}}({\rm{ \% }})={\rm{n}}{\rm{u}}{\rm{m}}{\rm{b}}{\rm{e}}{\rm{r}}\,{\rm{o}}{\rm{f}}\,{\rm{c}}{\rm{o}}{\rm{l}}{\rm{o}}{\rm{n}}{\rm{i}}{\rm{e}}{\rm{s}}\,{\rm{o}}{\rm{f}}\,A.\,terreus\,{\rm{o}}{\rm{n}}\,{\rm{t}}{\rm{h}}{\rm{e}}\,{\rm{a}}{\rm{g}}{\rm{a}}{\rm{r}}\,{\rm{m}}{\rm{e}}{\rm{d}}{\rm{i}}{\rm{a}}\,/{\rm{n}}{\rm{u}}{\rm{m}}{\rm{b}}{\rm{e}}{\rm{r}}\,{\rm{o}}{\rm{f}}\,{\rm{c}}{\rm{o}}{\rm{l}}{\rm{o}}{\rm{n}}{\rm{i}}{\rm{e}}{\rm{s}}\,{\rm{o}}{\rm{f}}\,{\rm{s}}{\rm{o}}{\rm{i}}{\rm{l}}\,{\rm{m}}{\rm{i}}{\rm{c}}{\rm{r}}{\rm{o}}{\rm{b}}{\rm{e}}{\rm{s}}\,{\rm{o}}{\rm{n}}\,{\rm{t}}{\rm{h}}{\rm{e}}\,{\rm{a}}{\rm{g}}{\rm{a}}{\rm{r}}\,{\rm{m}}{\rm{e}}{\rm{d}}{\rm{i}}{\rm{a}}.\end{array}$$

### Isolation of microbes producing vinyl compounds from soil samples using DISCOVER

The thiol-ene reaction (1^st^ step of screening) was performed as follows. Three microliters of the soil supernatants were spotted onto PD and GM1 agar media supplemented with 50 mM TG and 0.5 mM VA, followed by incubation at 30 °C for 1 to 3 weeks until colonies appeared. The colonies obtained were picked using a toothpick and then inoculated into 700 μL of GM2 medium in a 96 well deep plate (volume, 1.1 mL) covered with sealing tape. The cultures were incubated at 30 °C for 7 days using an incubator M·BR-034P (TAITEC, Saitama, Japan) with shaking at 1600 rpm. The Mizoroki-Heck reaction followed by iodine test (2^nd^ step of screening) was performed according to the method described elsewhere^17^. Mass determination of vinyl compounds labelled with IB was performed in the same manner as analysis of the reactants of TG with IA.

### Sequence analysis of 26S rRNA gene

Genomic DNA was extracted from the harvested cells using Cica genus DNA extraction reagent ST (Kanto Chemical Co., Inc., Tokyo, Japan). PCR was performed using the extracted genomic DNA as a template and the following set of primers: NL1, 5′-GCATATCAATAAGCGGAGGAAAAG-3′ and NL4, 5′-GGTCCGTGTTTCAAGACGG-3′ for the D1/D2 region in the 26S rRNA gene, with KOD plus DNA polymerase (Toyobo, Shiga, Japan), in the procedures outlined by the manufacturer. The following conditions were employed for the PCR: denaturation at 94 °C for 20 s, annealing at 50 °C for 15 s, and elongation at 68 °C for 30 s, continuing for 30 cycles. PCR products were purified and then sequenced by SolGent Co., Ltd. (Daejeon, Korea). The sequence similarity searches were performed with the BLAST (Basic Local Alignment Search Tool) program and the NCBI (National Center for Biotechnology Information) databases.

## Supplementary information

Figure S1

## References

[1] Wei Z (2013). Phenotypic screens as a renewed approach for drug discovery. Drug Discov. Today.

[2] Mounyr B (2016). Methods for *in vitro* evaluating antimicrobial activity: A review. J. Pharm. Anal..

[3] Calam CT (1939). Studies in the biochemistry of micro-organisms: Itaconic acid, a metabolic product of a strain of *Aspergillus terreus* Thom. Biochem. J..

[4] Kuenz A (2012). Microbial production of itaconic acid: Developing a stable platform for high product concentrations. Appl. Microbiol. Biotechnol..

[5] Kinoshita K (1931). Über eine neue Aspergillus-Art, Asp. itaconicus nov. spec. Shokubutsugaku Zasshi..

[6] Guevarra ED, Tabuchi T (1990). Accumulation of itaconic, 2-hydroxyparaconic, itatartaric, and malic acids by strains of the genus ustilago. Agric. Biol. Chem..

[7] Tabuchi T (1981). Itaconic acid fermentation by a yeast belonging to the genus candida. Agric. Biol. Chem..

[8] Levinson WE (2006). Production of itaconic acid by *Pseudozyma antarctica* NRRL Y-7808 under nitrogen-limited growth conditions. Enzyme Microb. Technol..

[9] Klement T, Buchs J (2013). Itaconic acid - A biotechnological process in change. Bioresour. Technol..

[10] Okabe M (2009). Biotechnological production of itaconic acid and its biosynthesis in *Aspergillus terreus*. Appl. Microbiol. Biotechnol..

[11] Willke T, Vorlop KD (2001). Biotechnological production of itaconic acid. Appl. Microbiol. Biotechnol..

[12] Andrew BL (2010). Thiol-ene “click” reactions and recent applications in polymer and materials synthesis. Polym. Chem..

[13] Knowles JP, Whiting A (2007). The Heck-Mizoroki cross-coupling reaction: a mechanistic perspective. Org. Biomol. Chem..

[14] Mizoroki T (1971). Arylation of olefin with aryl iodide catalyzed by palladium. Bull. Chem Soc. Jpn..

[15] Heck KF, Nolley JP (1972). Palladium-catalyzed vinylic hydrogen substitution reactions with aryl, benzyl, and styryl halides. J. Org Chem..

[16] Mori K (1973). Arylation of olefin with iodobenzene catalyzed by palladium. Bull. Chem Soc. Jpn..

[17] Sano M (2019). A high-throughput screening method based on the Mizoroki-Heck reaction for isolating itaconic acid-producing fungi from soils. Heliyon..

[18] David, C. *Reagents for Radical and Radical Ion Chemistry* (John Wiley & Sons Ltd, 2008).

[19] Jensen KK, Javor GT (1981). Inhibition of *Escherichia coli* by thioglycerol. Antimicrob. Agents Chemother..

[20] Javor GT (1983). Inhibition of respiration of Escherichia coli by thioglycerol. Antimicrob. Agents Chemother..

[21] Robert LB (1986). Thioglycerol inhibition of growth and aflatoxin production in *Aspergillus parasiticus*. J. Gen. Microbiol..

[22] Gniazdowki M (1975). Influence of thiols on the inhibition of ribonucleic acid synthesis *in vitro* by a 1-nitro-9 aminoalkyl acridine derivative, C283. Mol. Pharmacol..

[23] Lallier R (1962). The effects of thiosorbitol and thioglycerol on the structure of the mitotic process of *Paracentrotus lividus* sea urchin eggs. J. Cell Biol..

[24] Schild HO (1970). Lack of antagonism between thioglycerol and an oxytocin analog not containing a disulfide bond. Br. J. Pharmacol..

[25] Vargaftig BB (1974). Inhibition of sulfhydryl agents of arachidonic acid-induced platelet aggregation and release of potential inflammatory substances. Prostaglandins.

[26] Ellman GL (1959). Tissue sulfhydryl groups. Arch. Biochem. Biophys..

