# Supplementary material for: DISCOVER: A facile structure-based screening method for vinyl compound producing microbes
Source: Sci Rep. 2019 Nov 5;9:16007. doi: 10.1038/s41598-019-52518-6 (PMC6831603; doi:10.1038/s41598-019-52518-6)
Supplement: Supplementary file 1 — Figure S1 [file 41598_2019_52518_MOESM1_ESM.docx]

DISCOVER: A facile structure-based screening method for vinyl compound producing microbes

Yuji Aso^1*^, Mei Sano^1^, Hikari Kuroda^1^, Hitomi Ohara^1^, Hiroshi Ando^2^, Keiji Matsumoto^2^

^1^ Department of Biobased Materials Science, Kyoto Institute of Technology, 1 Hashigami-cho, Matsugasaki, Sakyo-ku, Kyoto 606-8585, Japan

^2^ Corporate R&B Planning Department, Kaneka Corporation, 2-3-18 Nakanoshima, Kita-ku, Osaka 530-8288, Japan

***** Corresponding author: E-mail: aso@kit.ac.jp; Phone/fax: +81-(0)75-724-7694


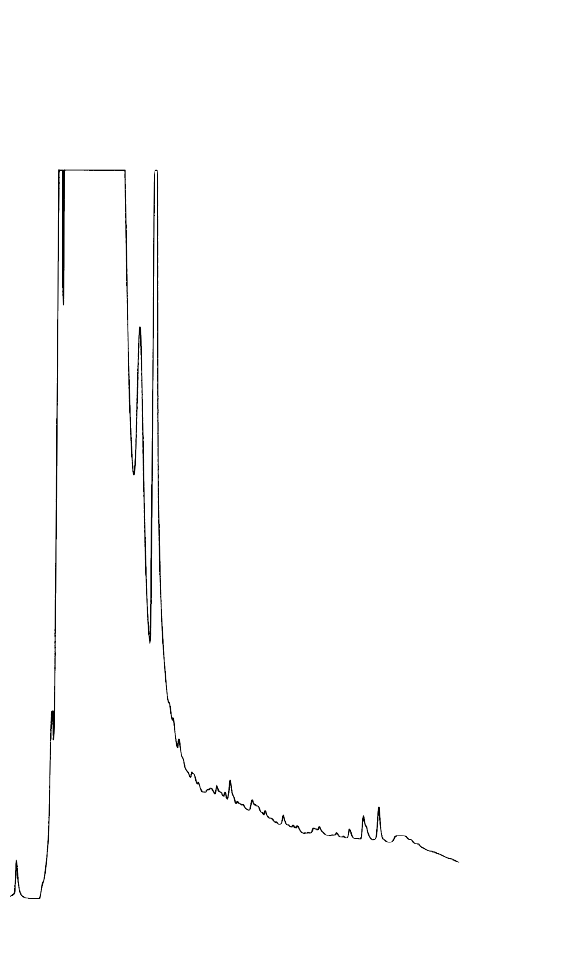

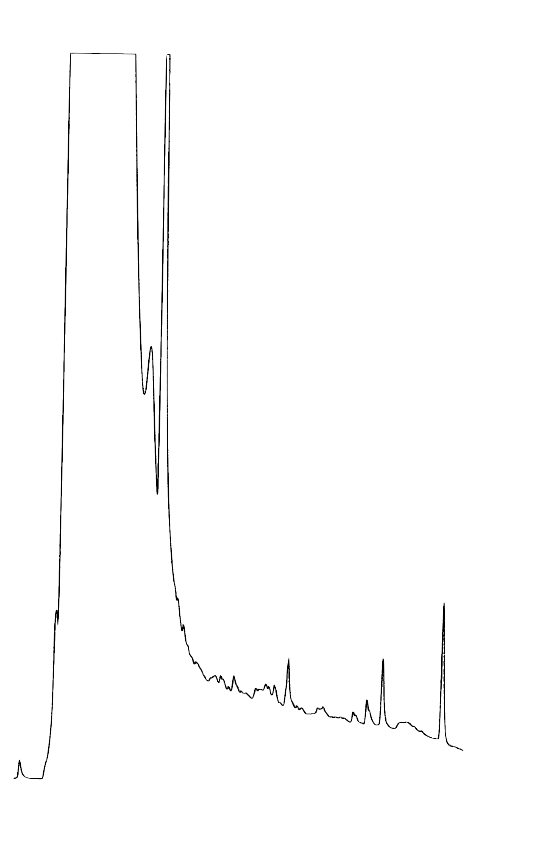

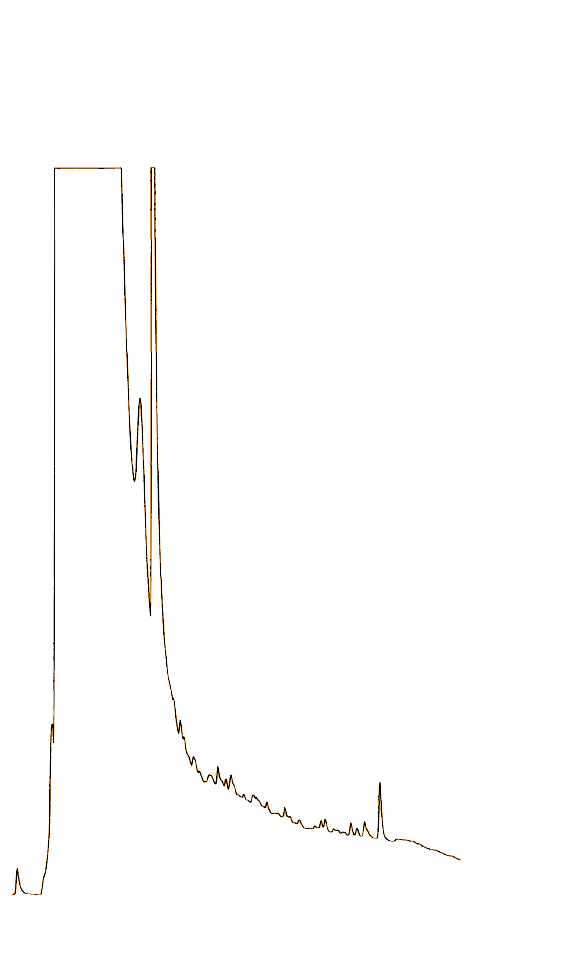

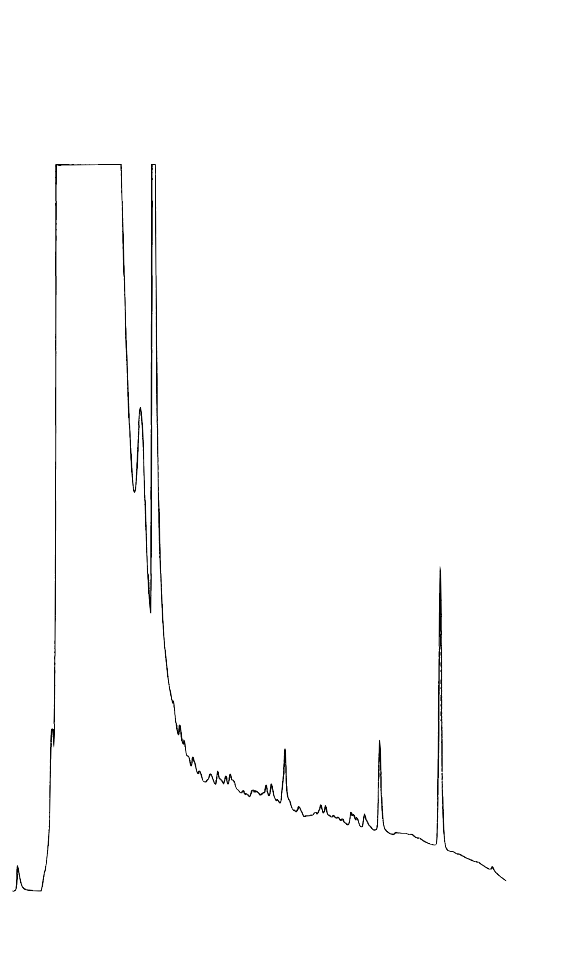
**Supplementary data**

(b)

(a)

(a)

(b)

A2-1


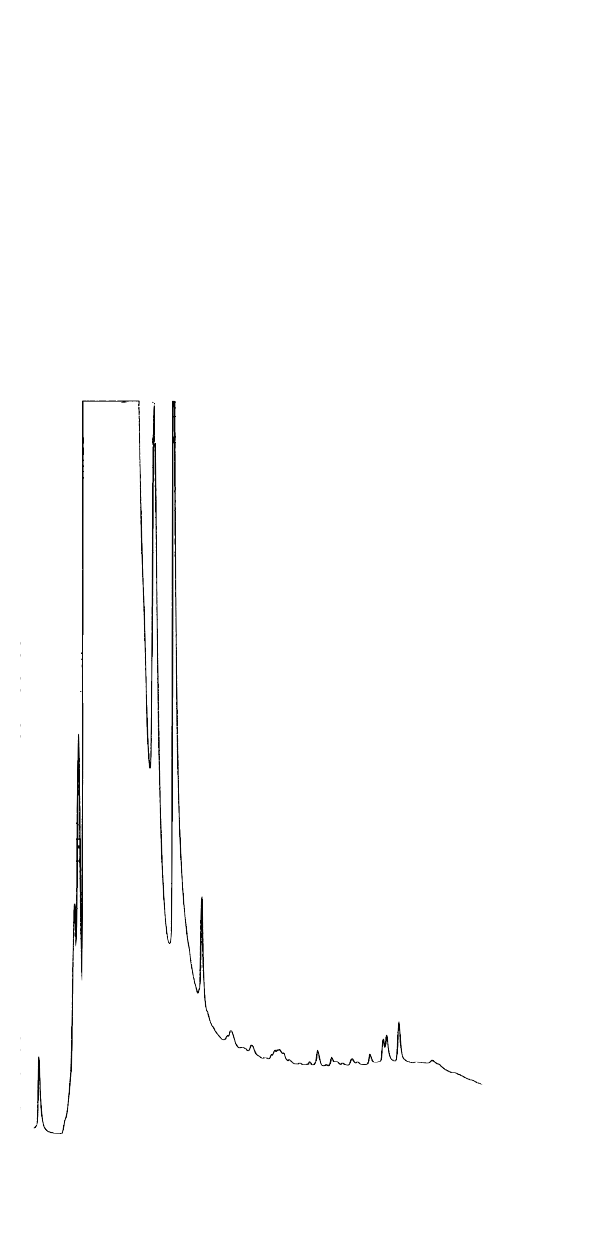


A10-3


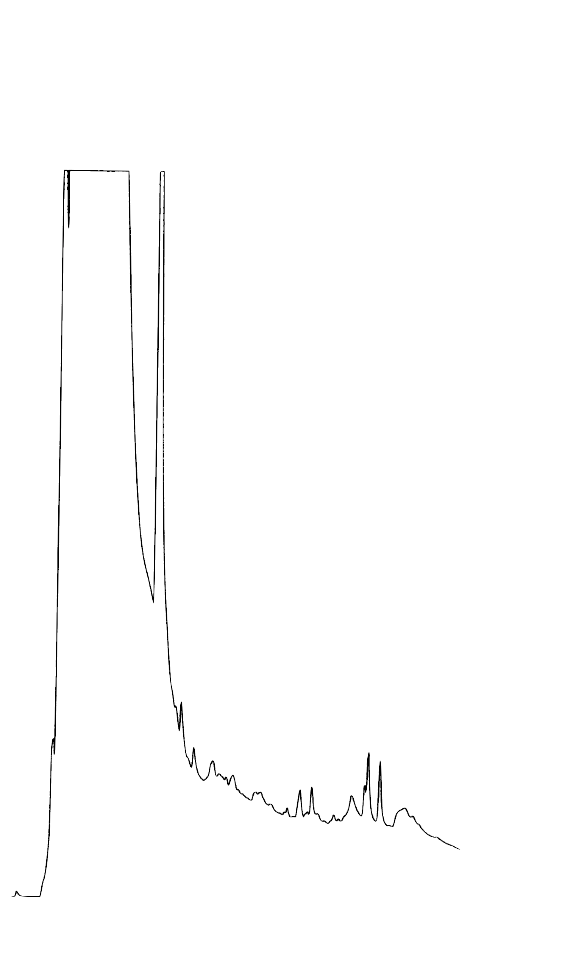

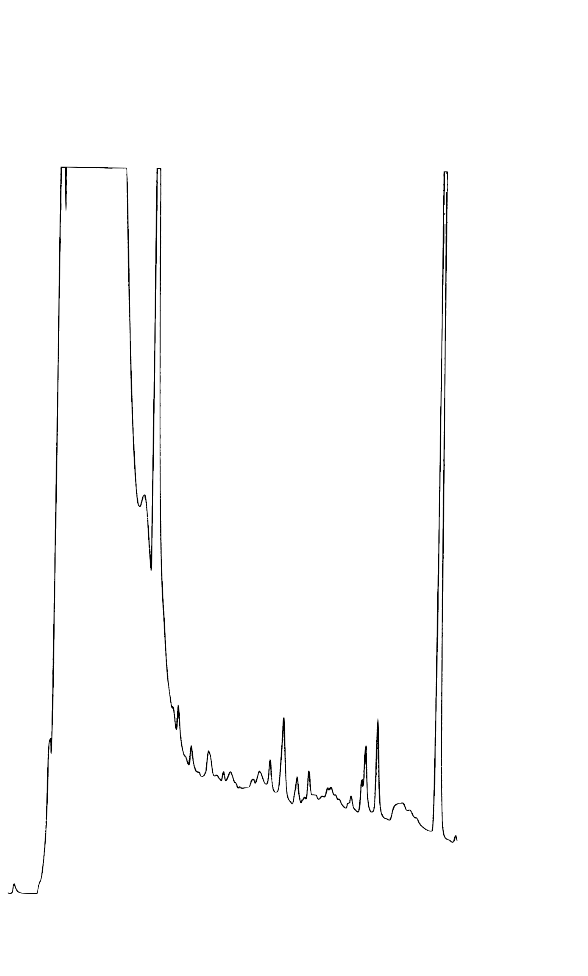

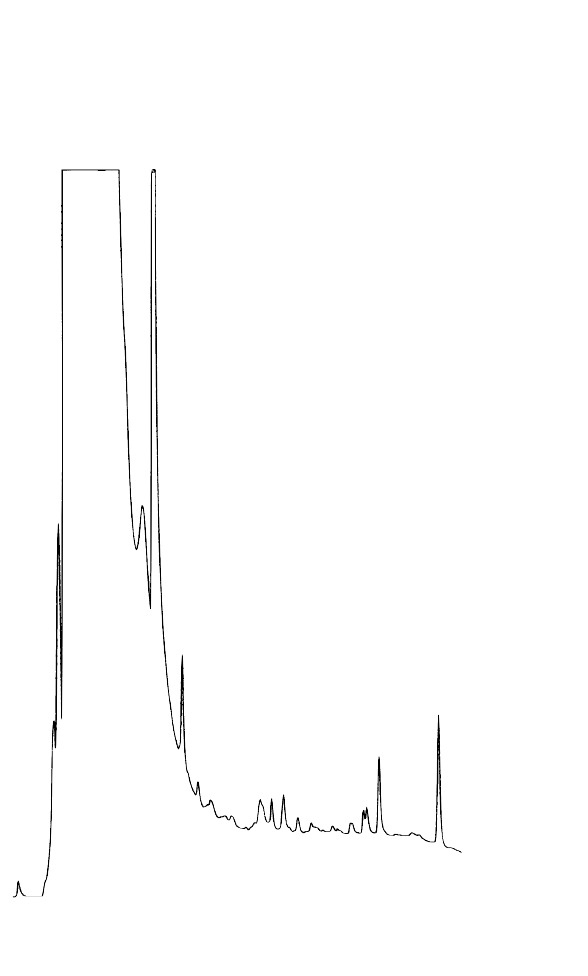


A4-1


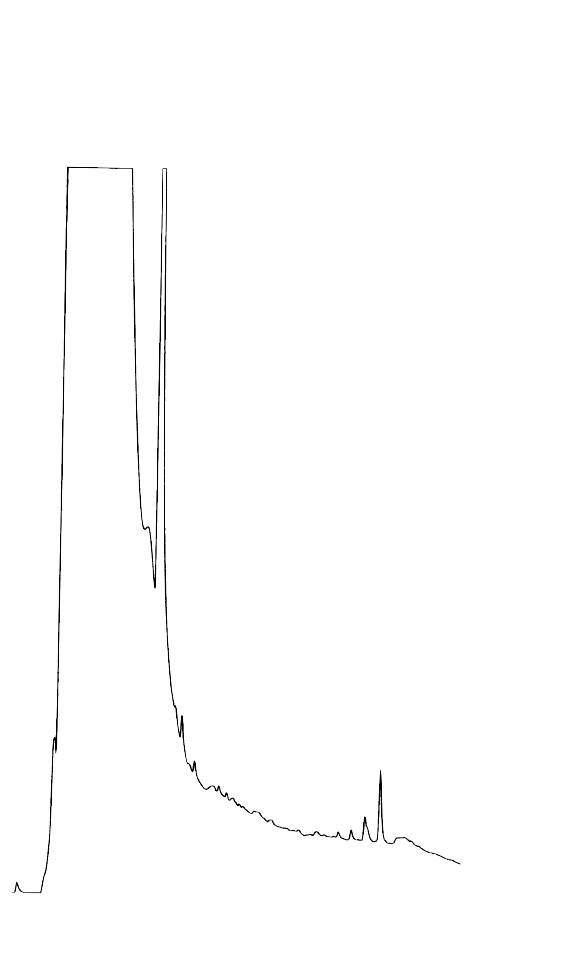

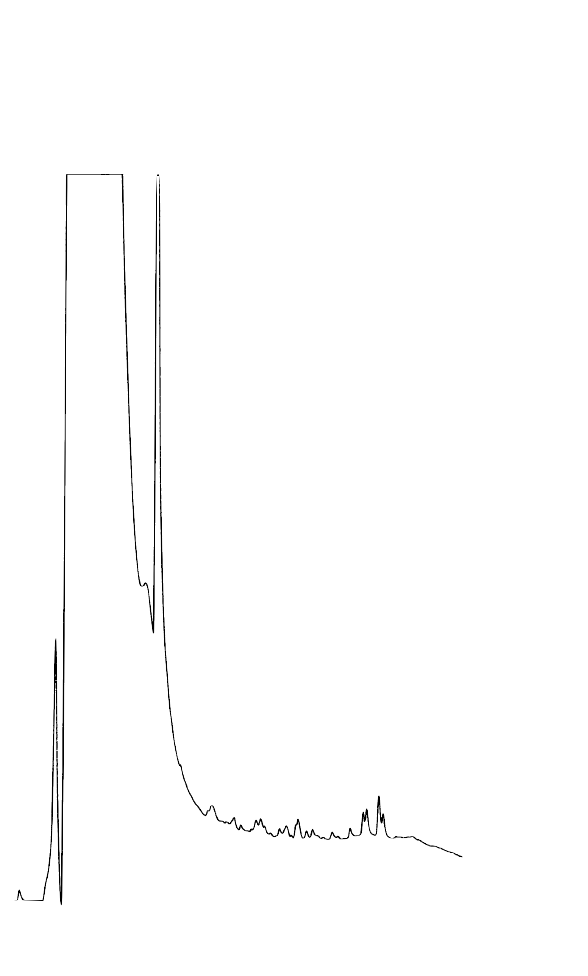

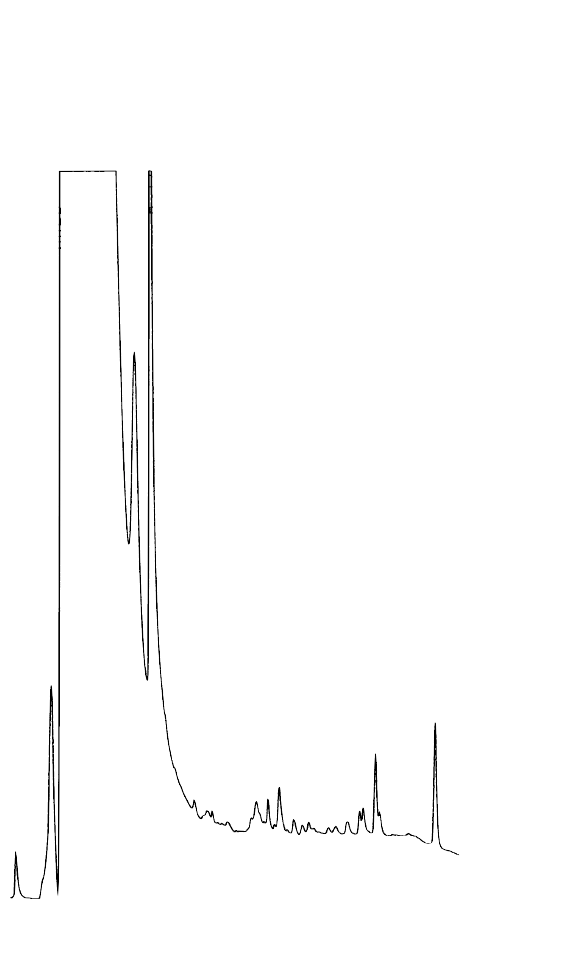


A11-1


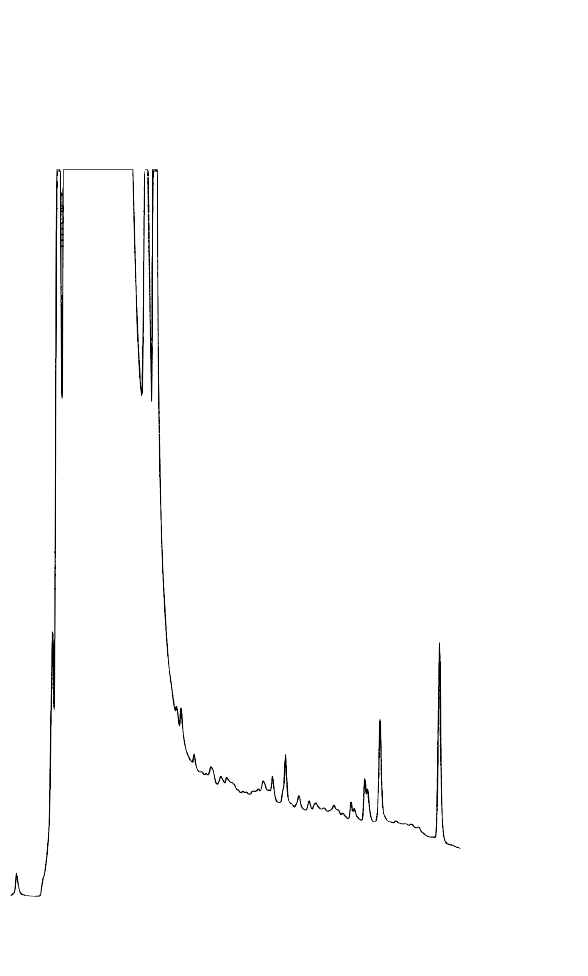


A4-2


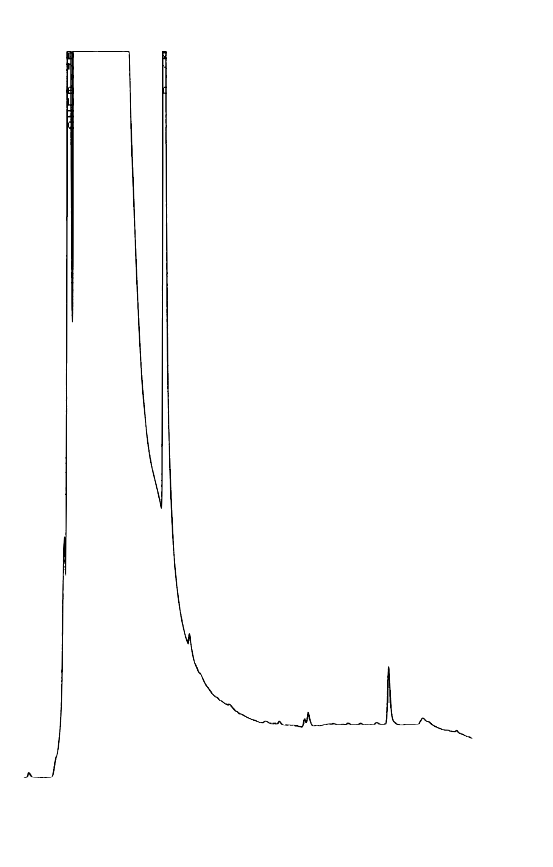

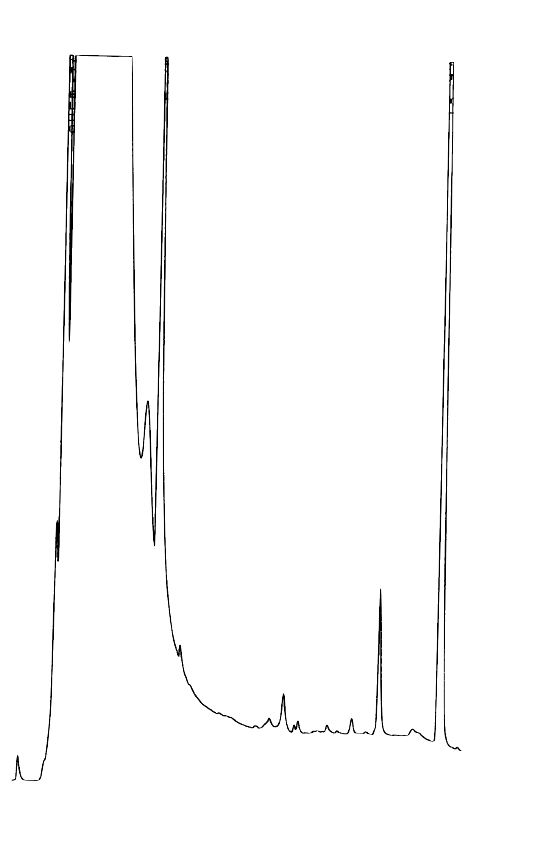

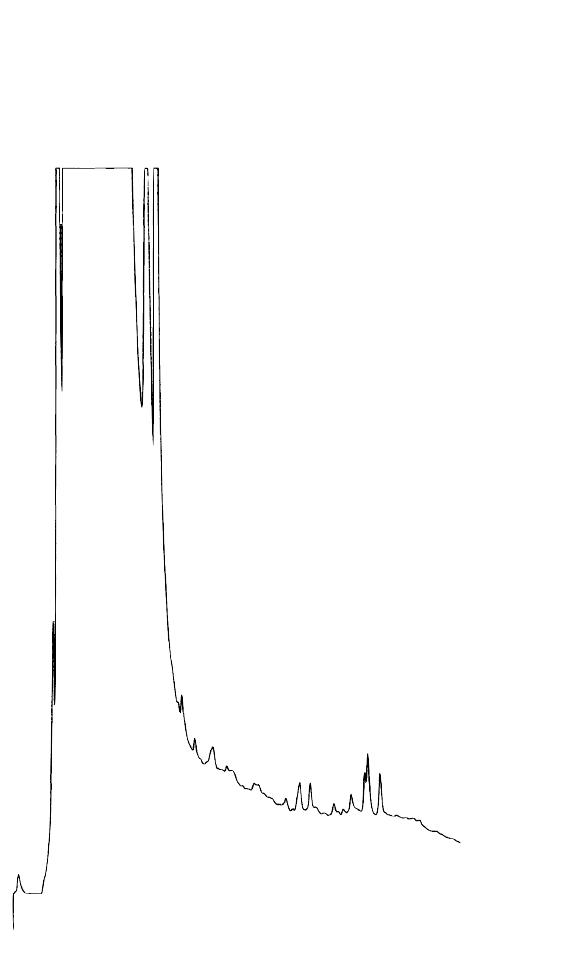

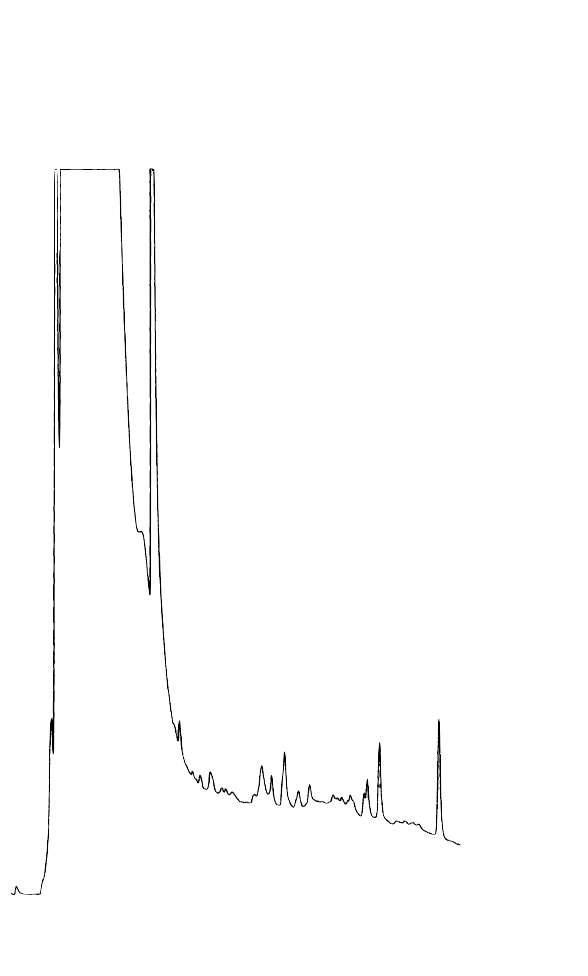


A16-1


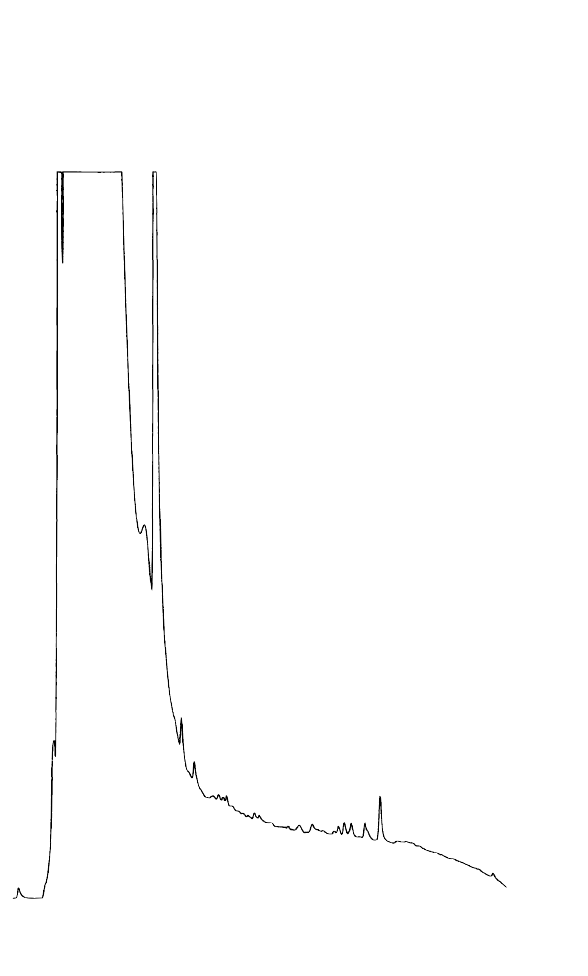


S5-1

A5-1


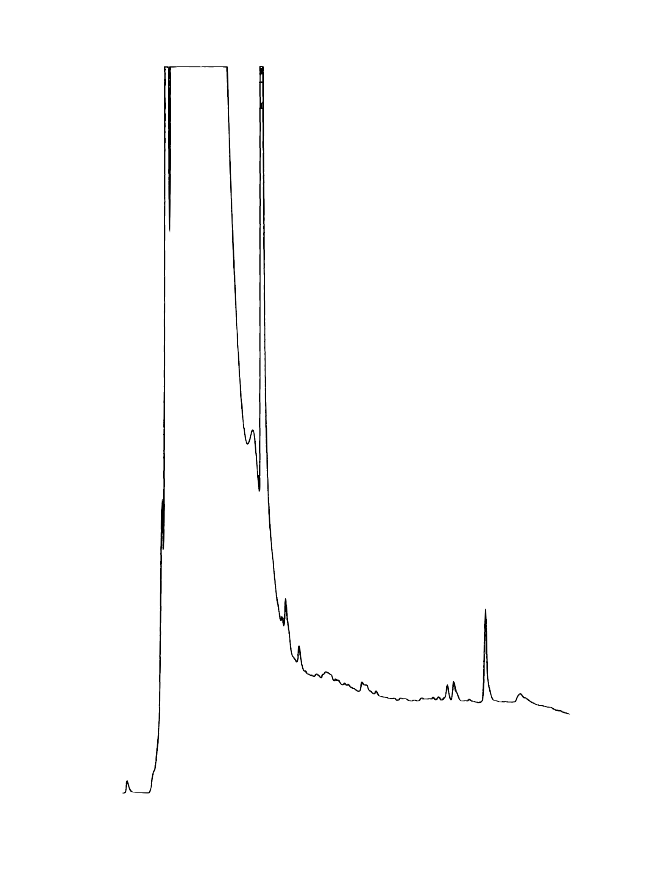

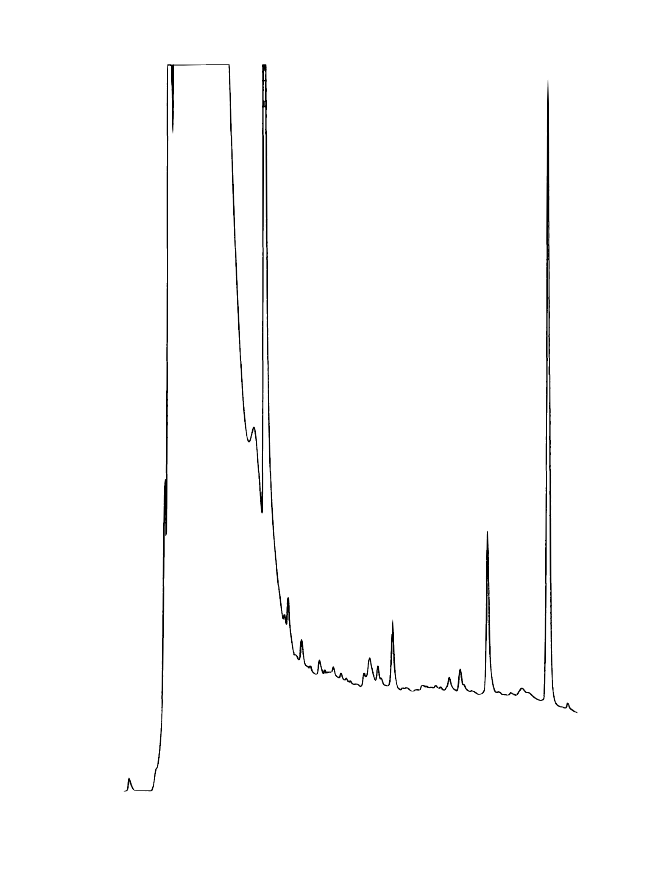

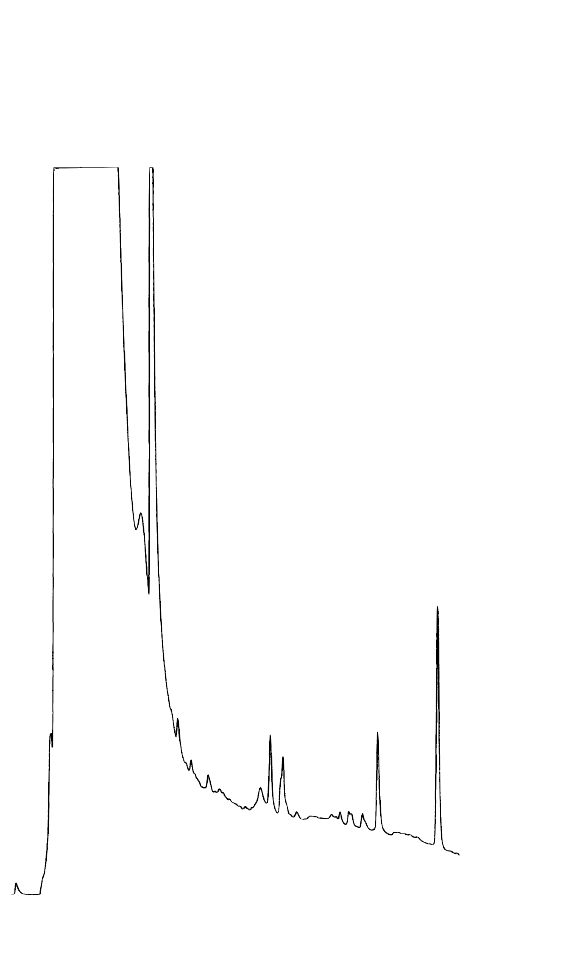


S9-1

A5-2


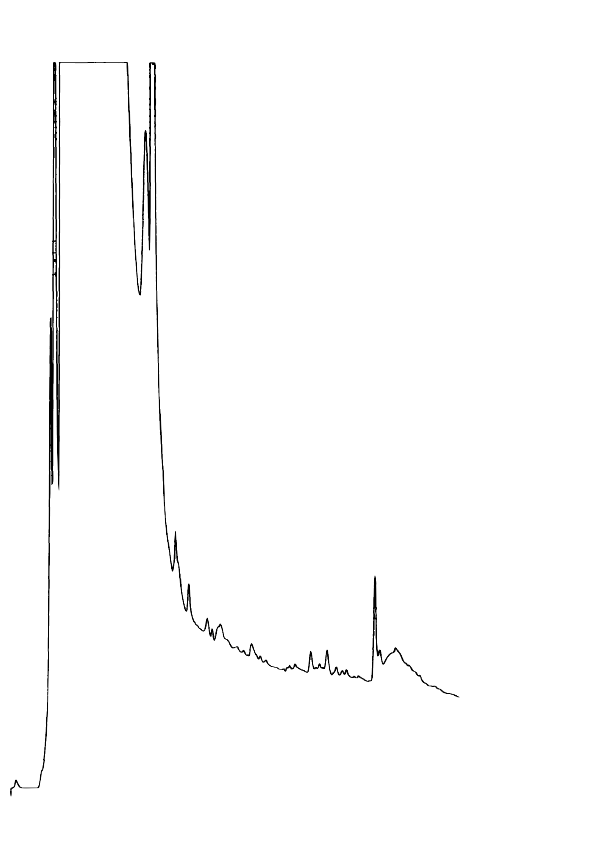

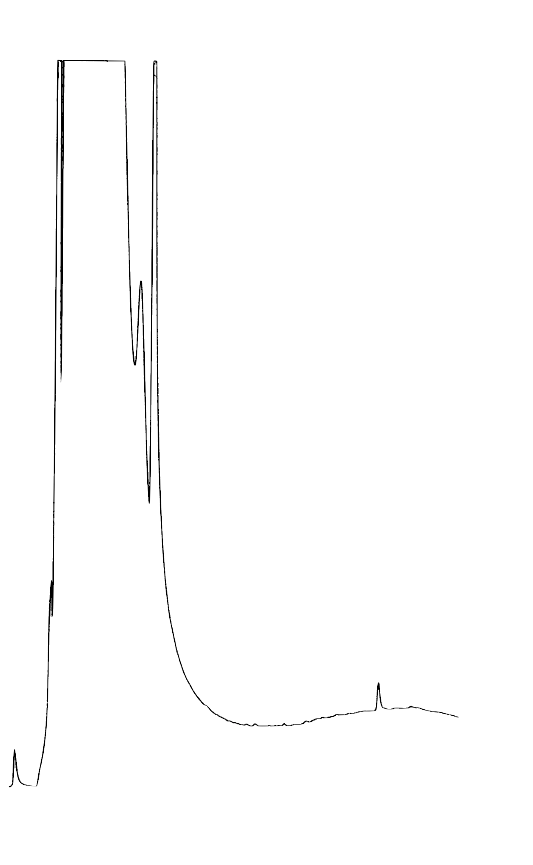

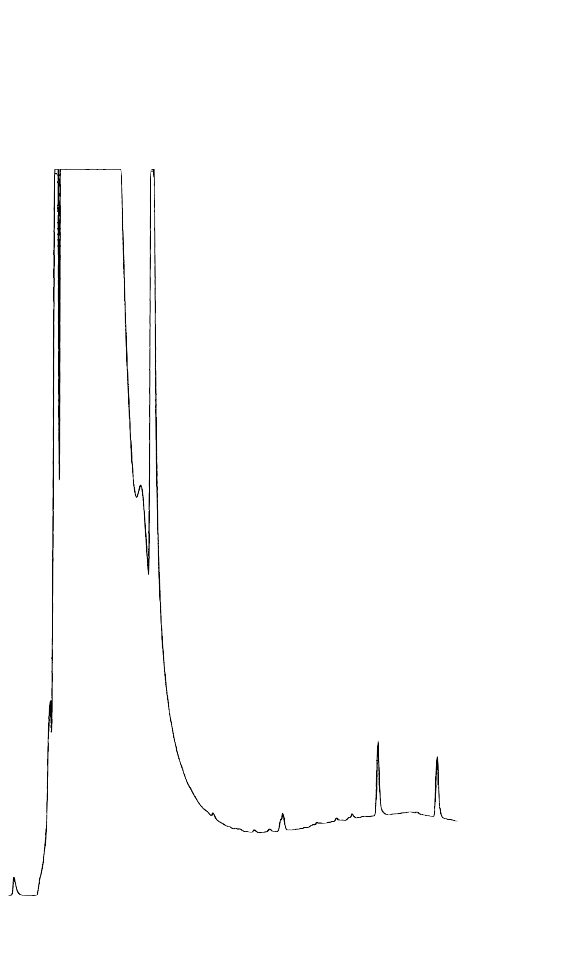


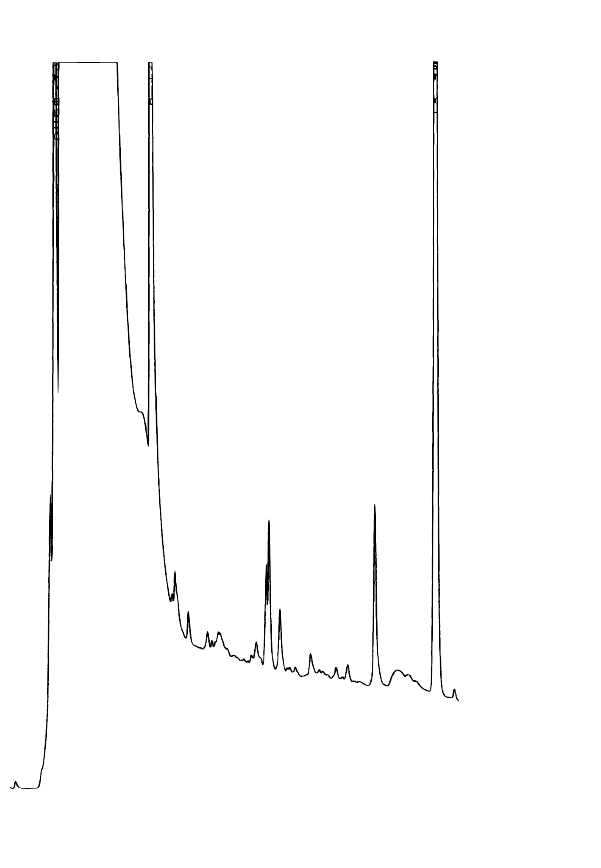


S12-1

A6-1


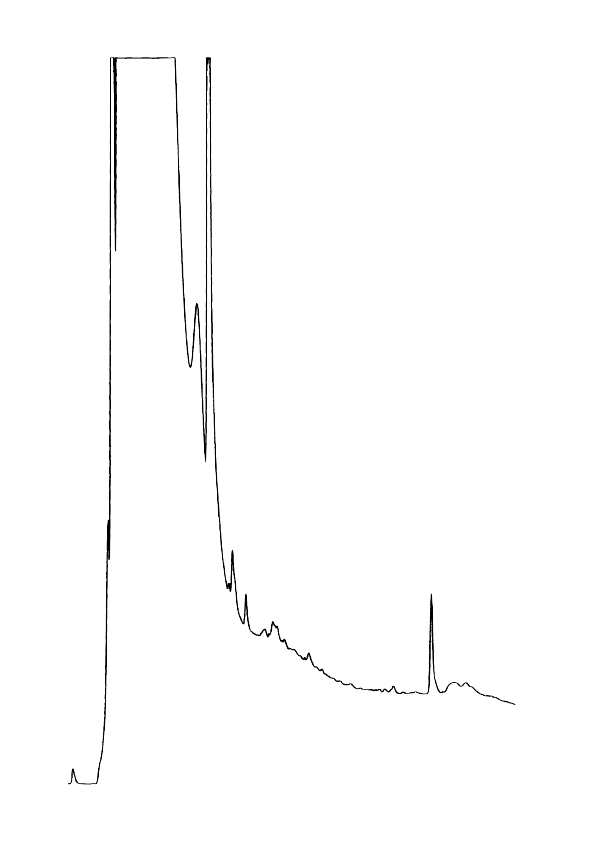

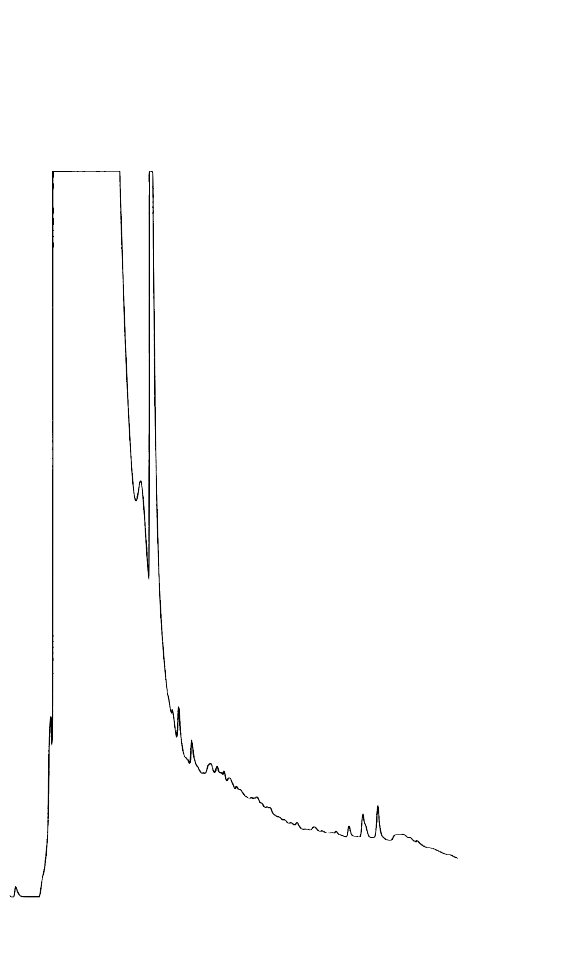

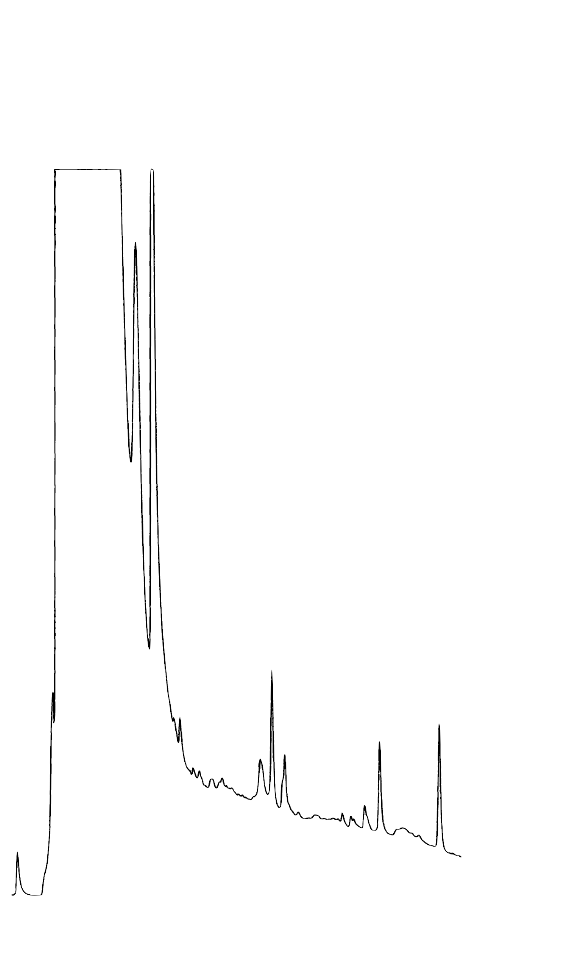


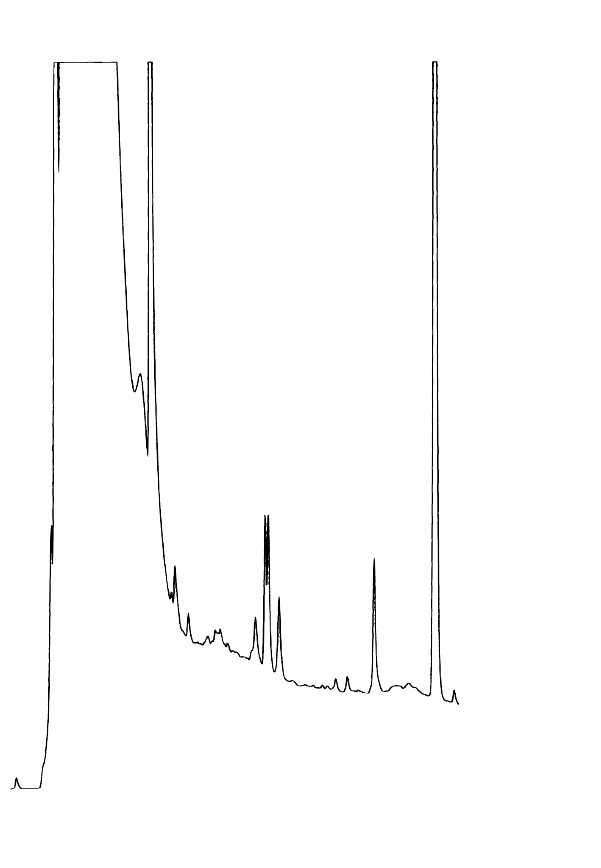


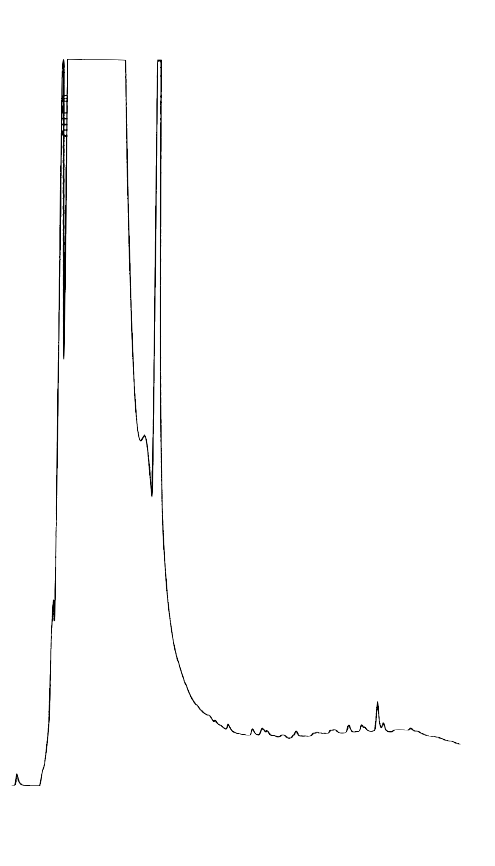

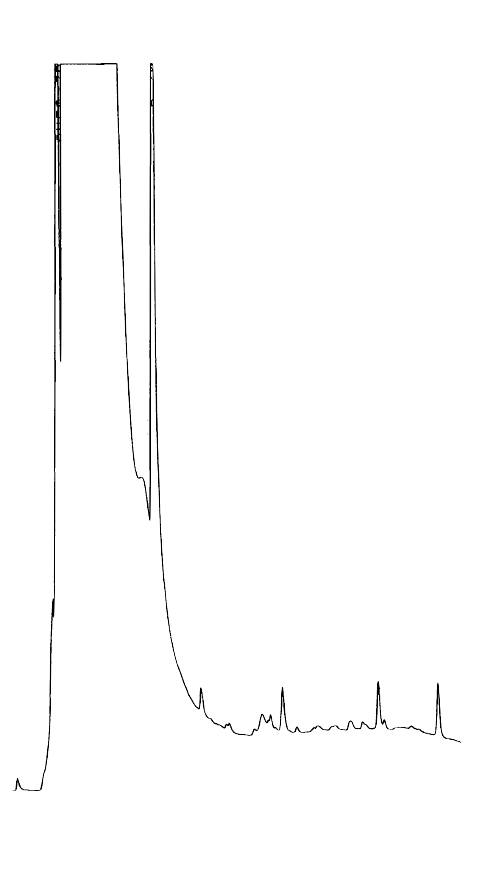


S12-2

A7-1


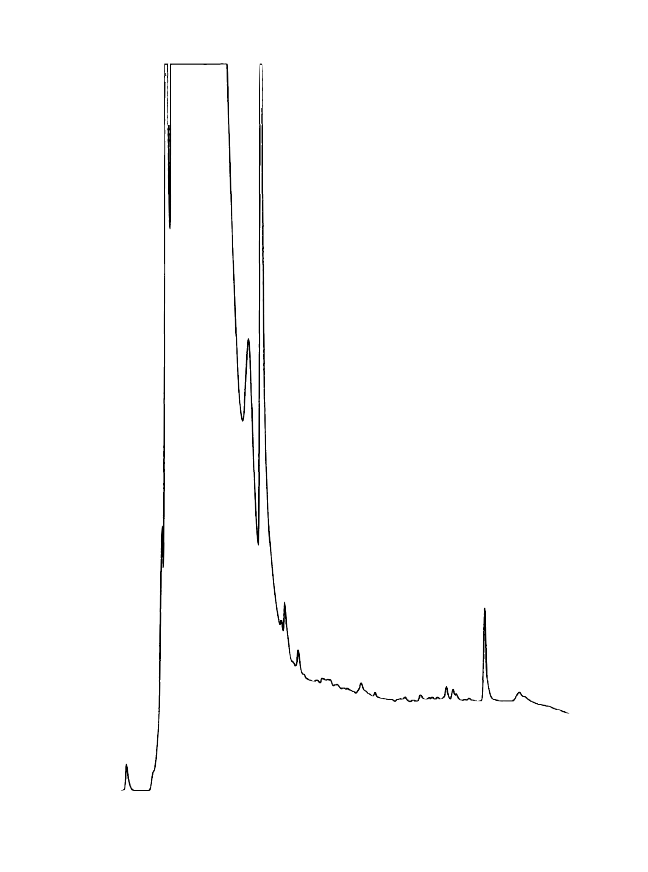

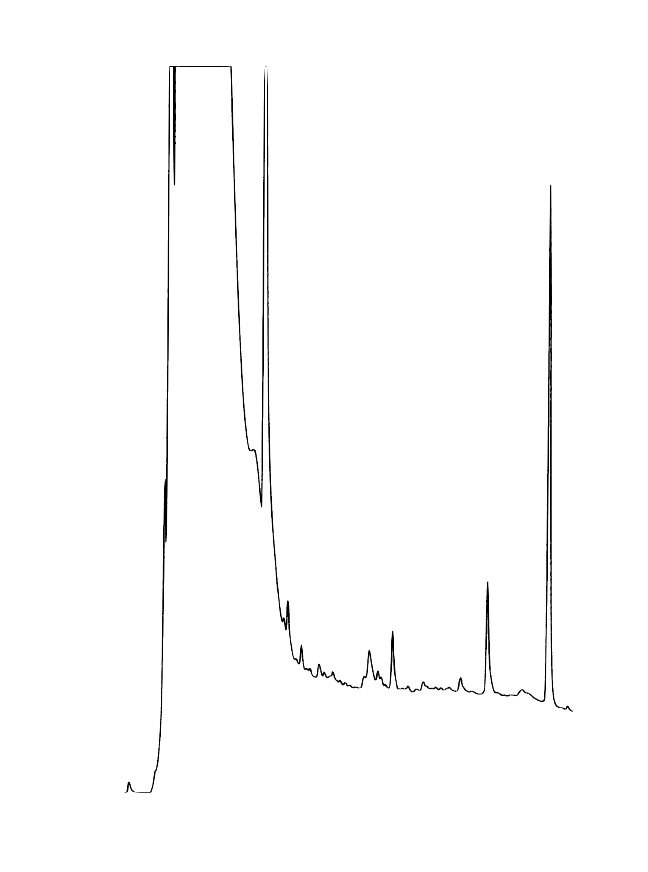


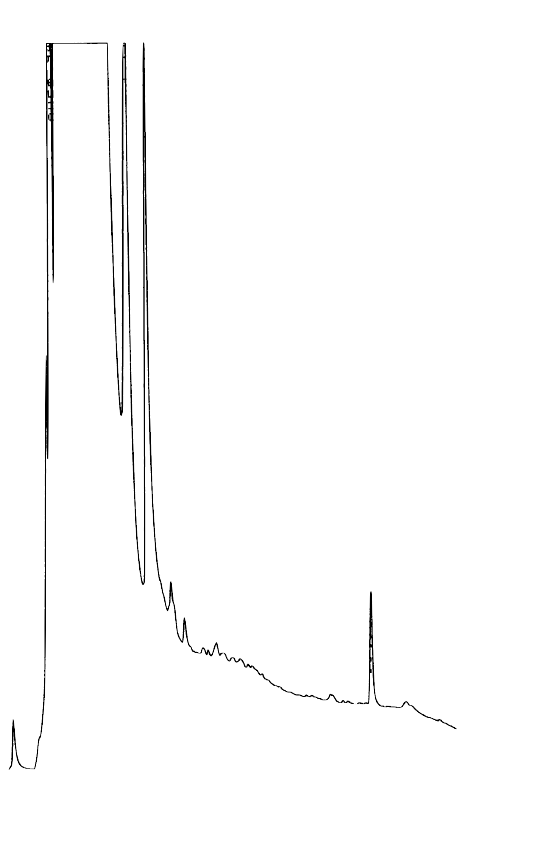

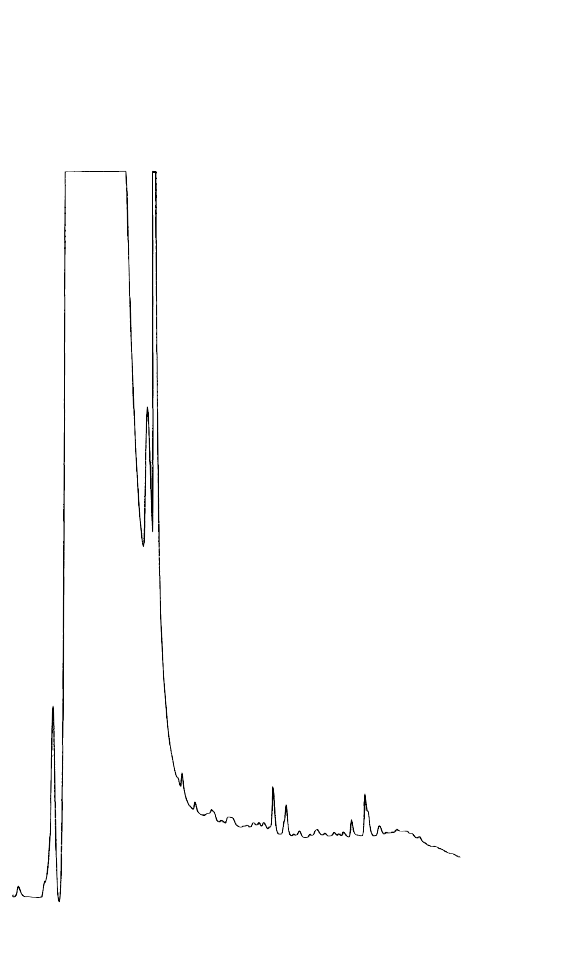

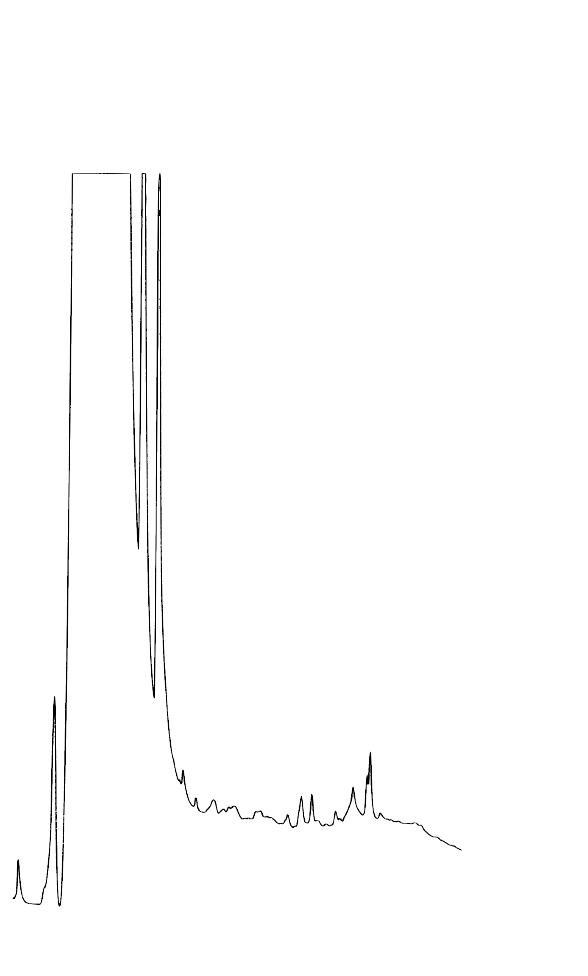


S12-3

A8-1


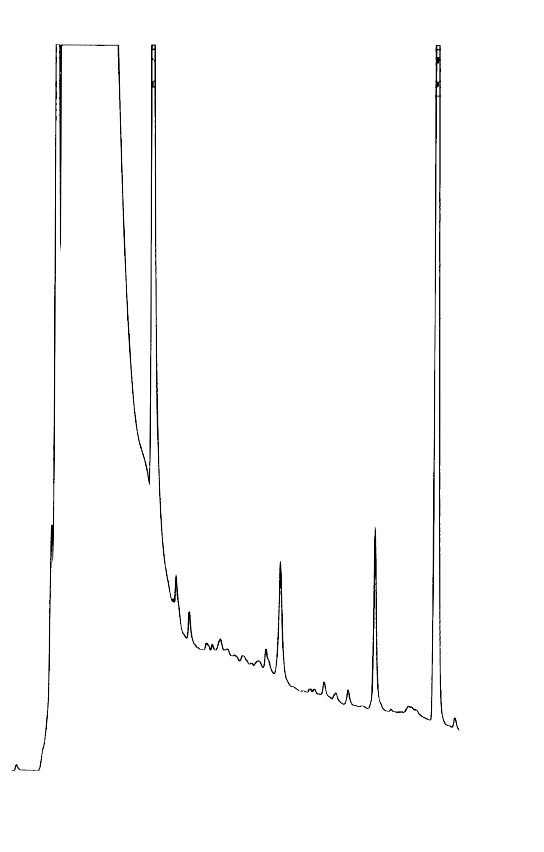


A10-1


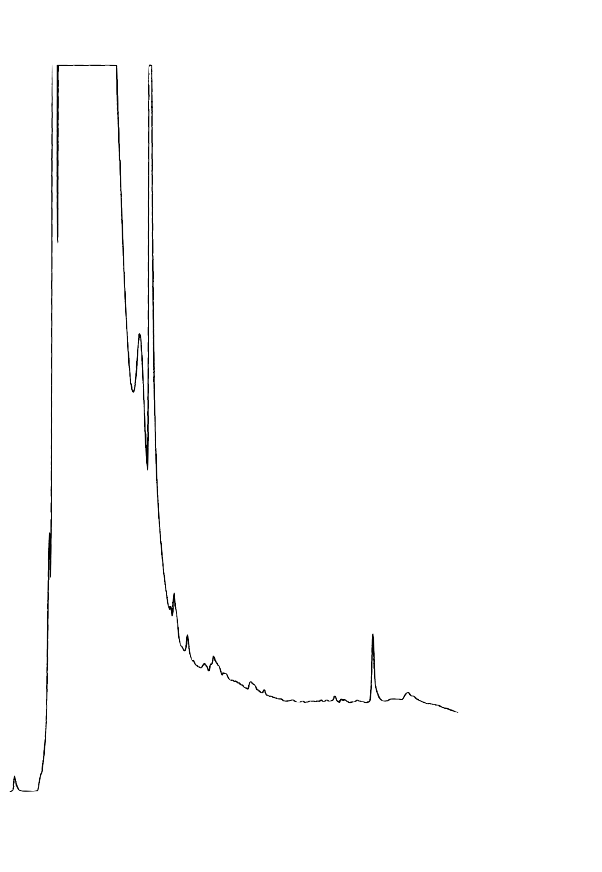

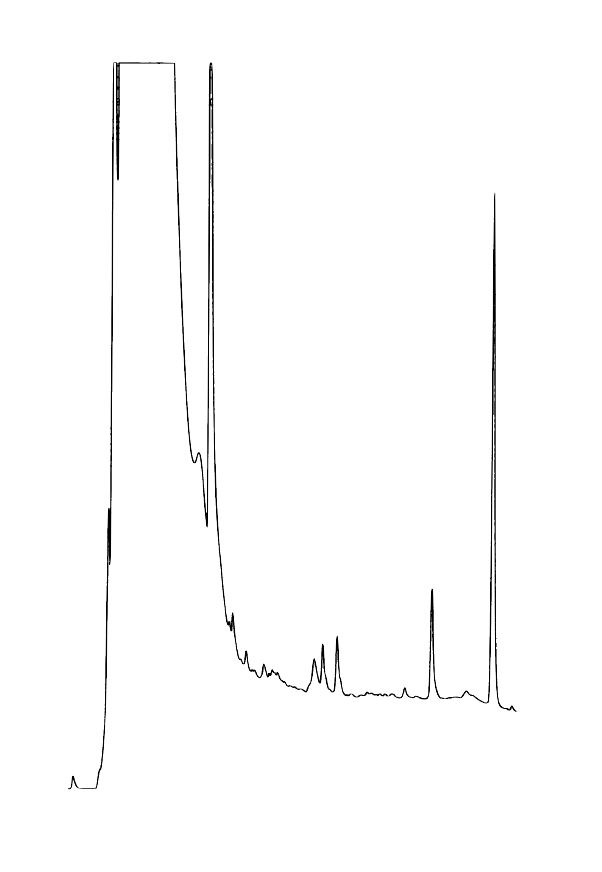

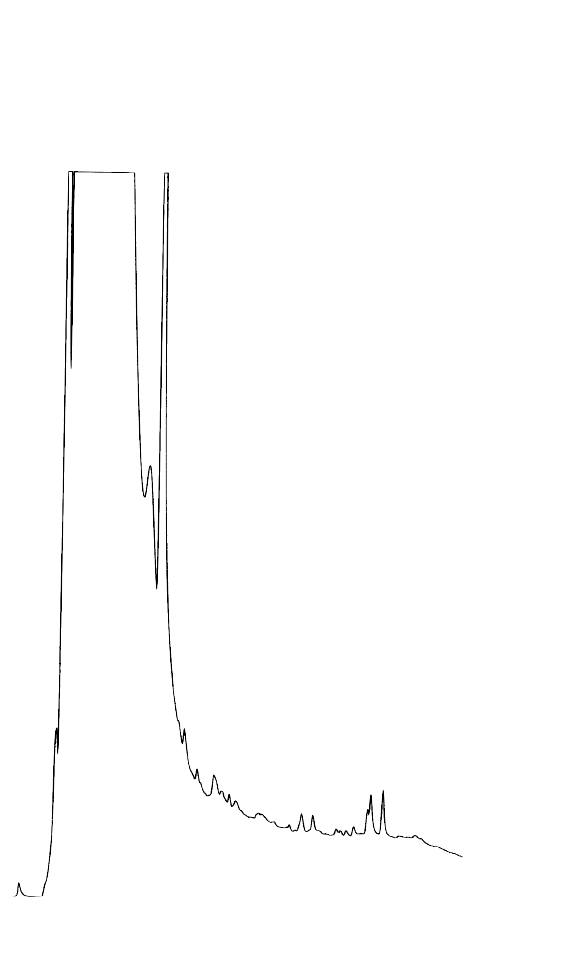

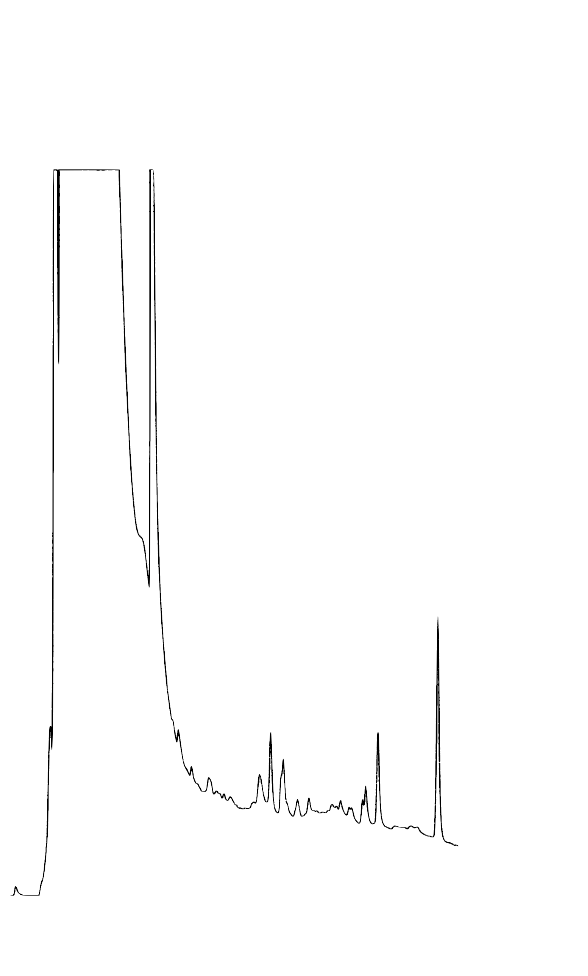


S13-1

S13-2

A10-2

8

10

15

20

(min)

8

10

15

20

(min)

8

10

15

20

(min)

8

10

15

20

(min)

**Figure S1**. HPLC chromatograms of the reactants after Mizoroki-Heck reaction with the cultures of the 45 strains in the (**a**) presence or (**b**) absence of iodobenzene (IB). The peaks corresponding to the vinyl compounds labelled with IB are marked by shadow.


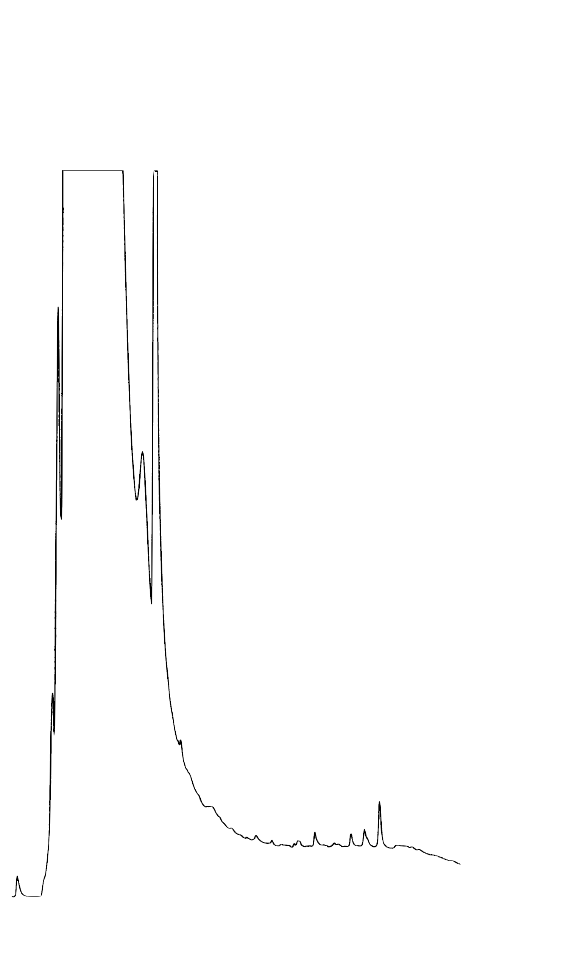

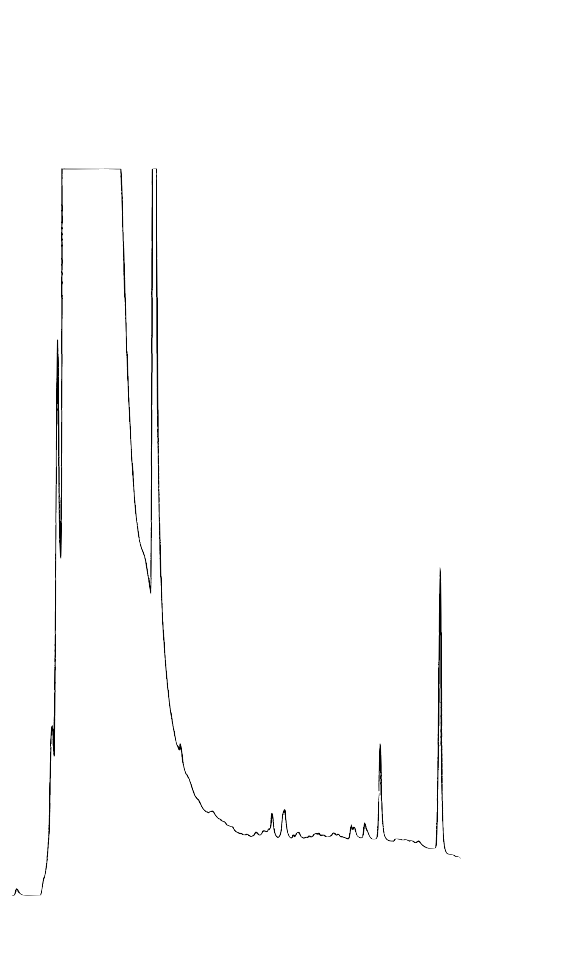

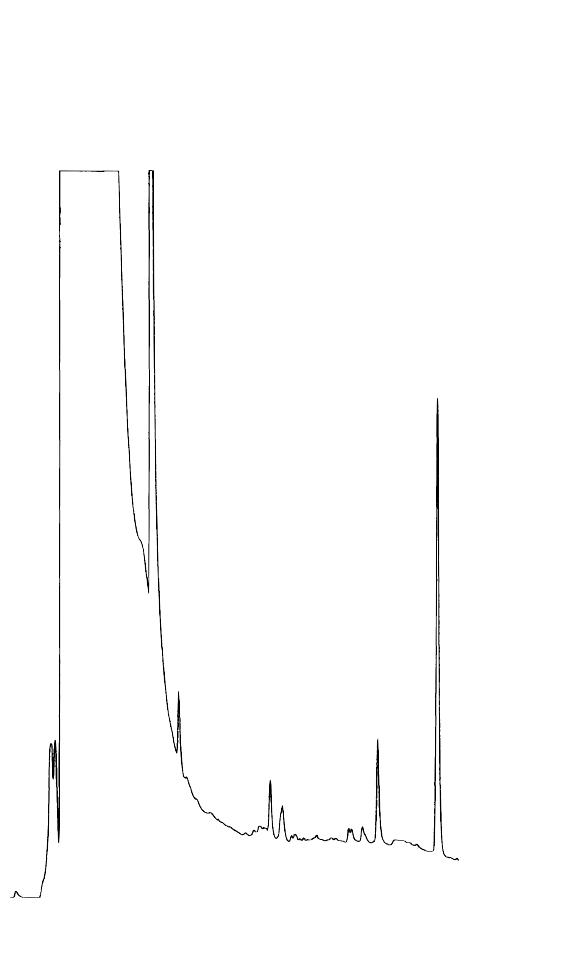

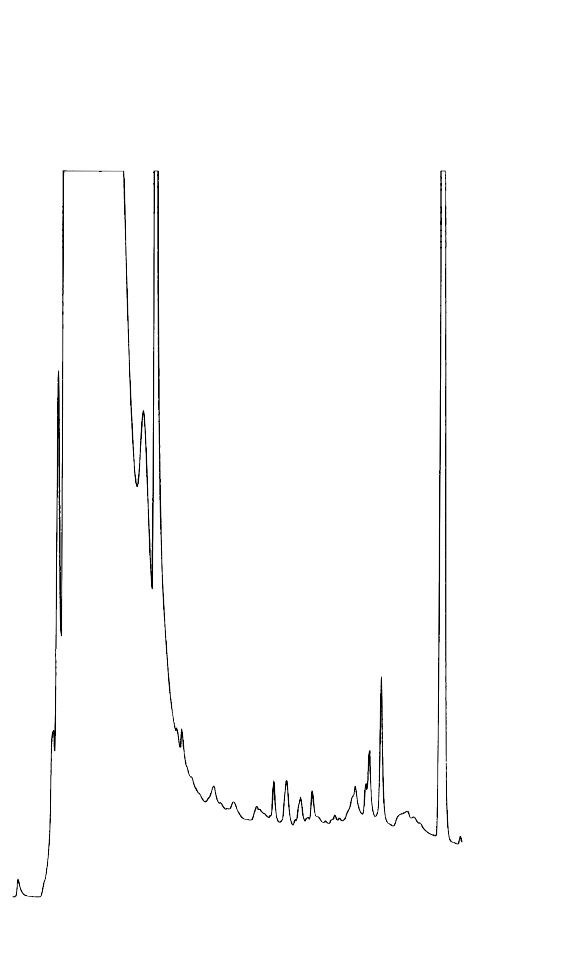

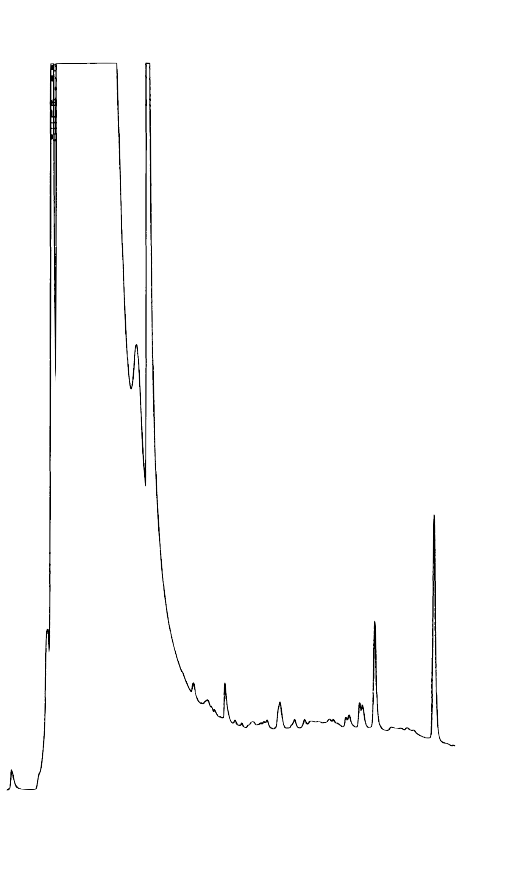

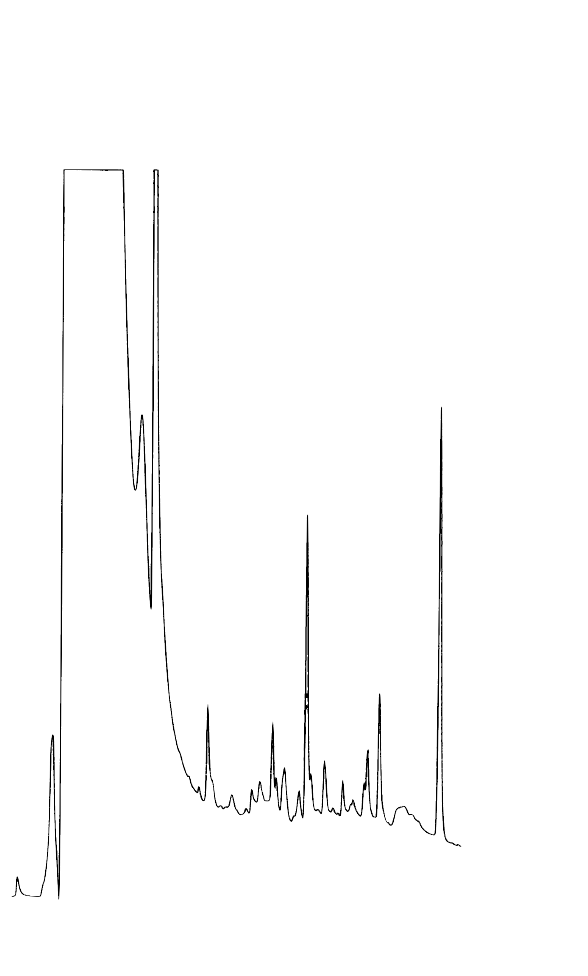

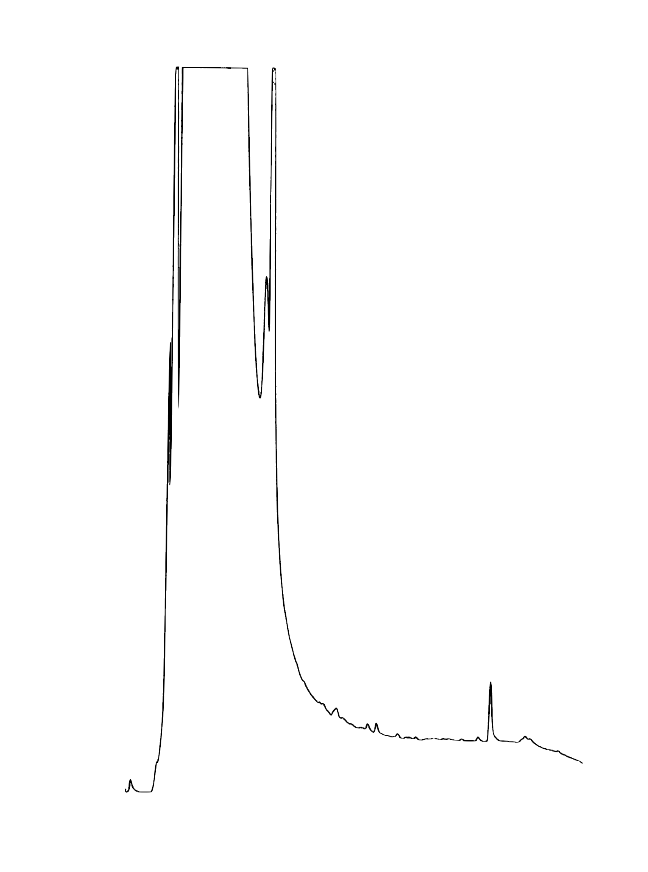

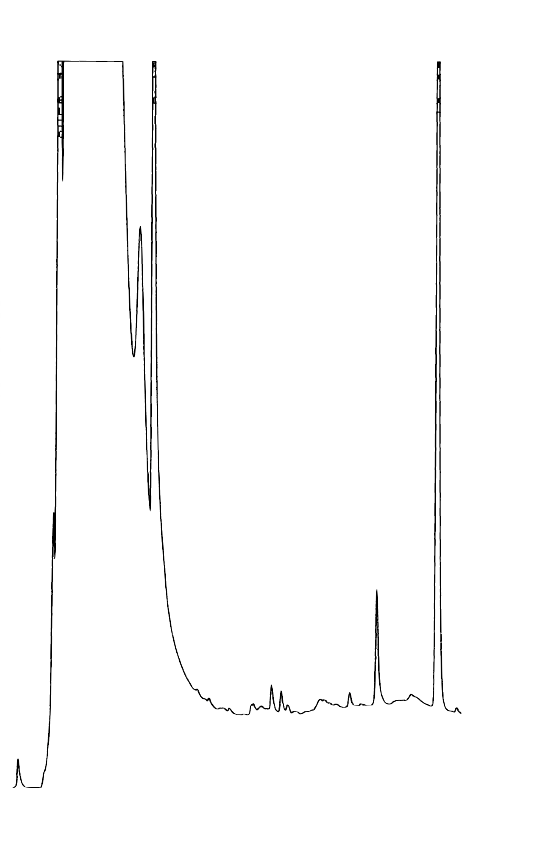

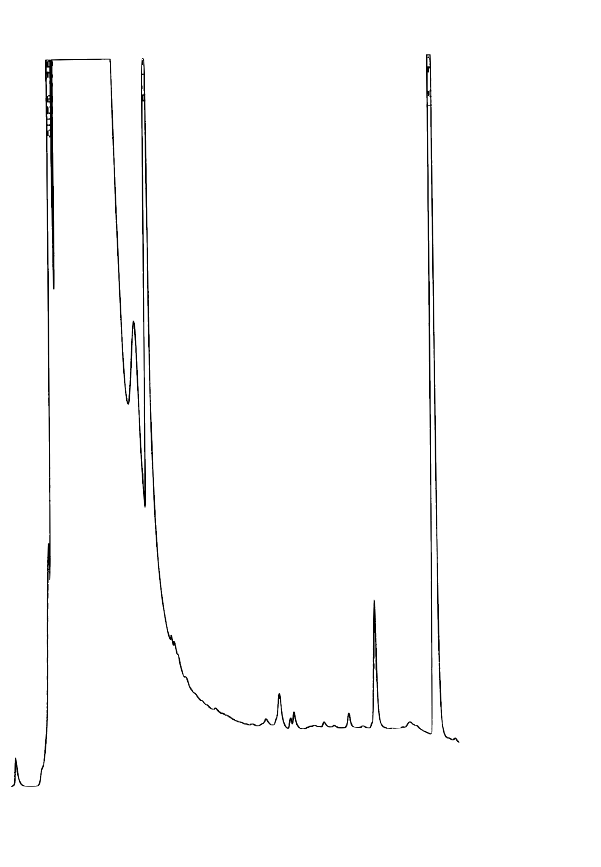

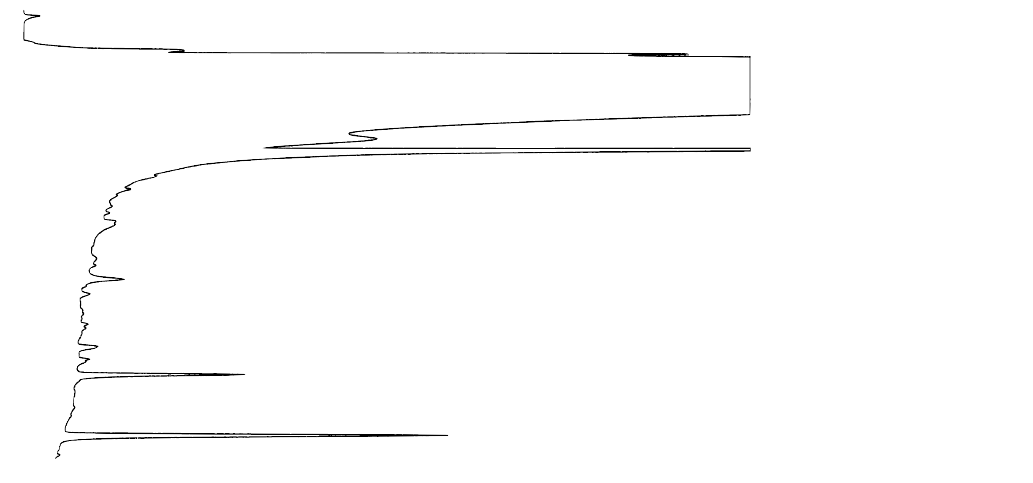

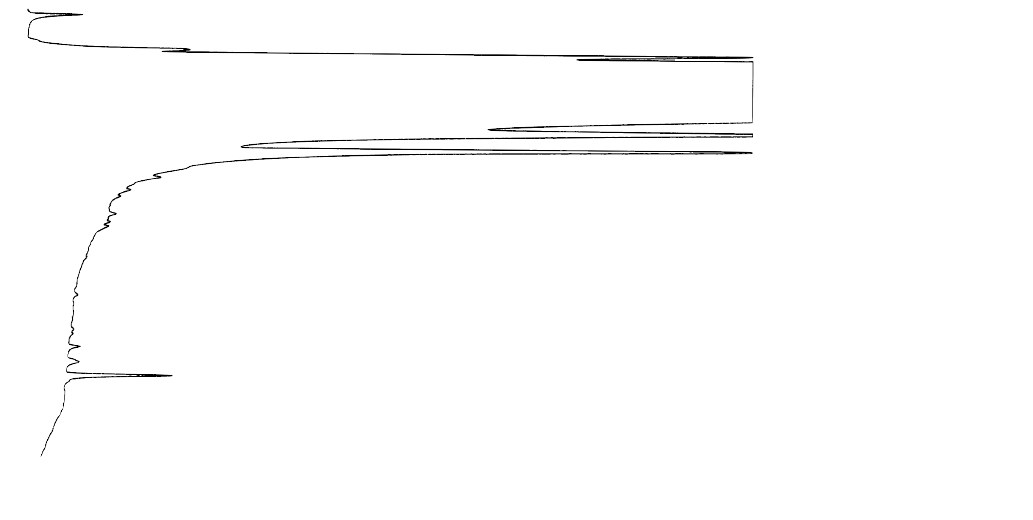


(a)

(b)

(a)

(b)

S14-1

8

10

15

20

(min)

S22-2

S23-1


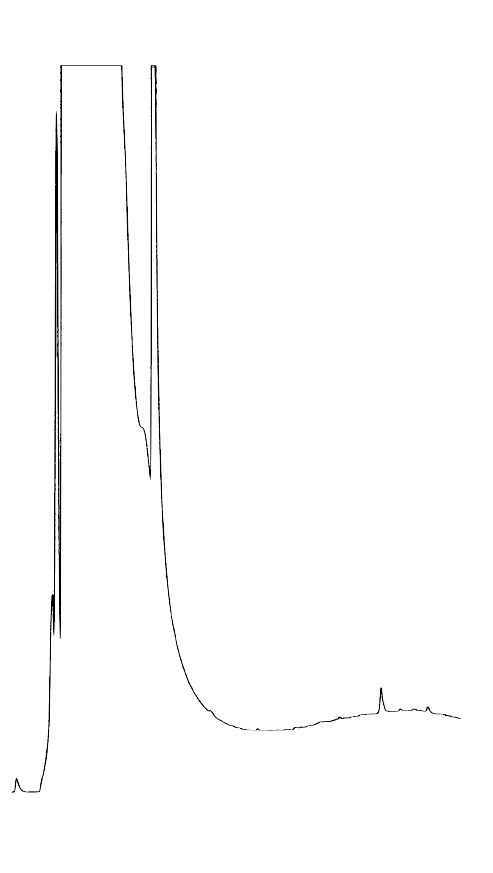

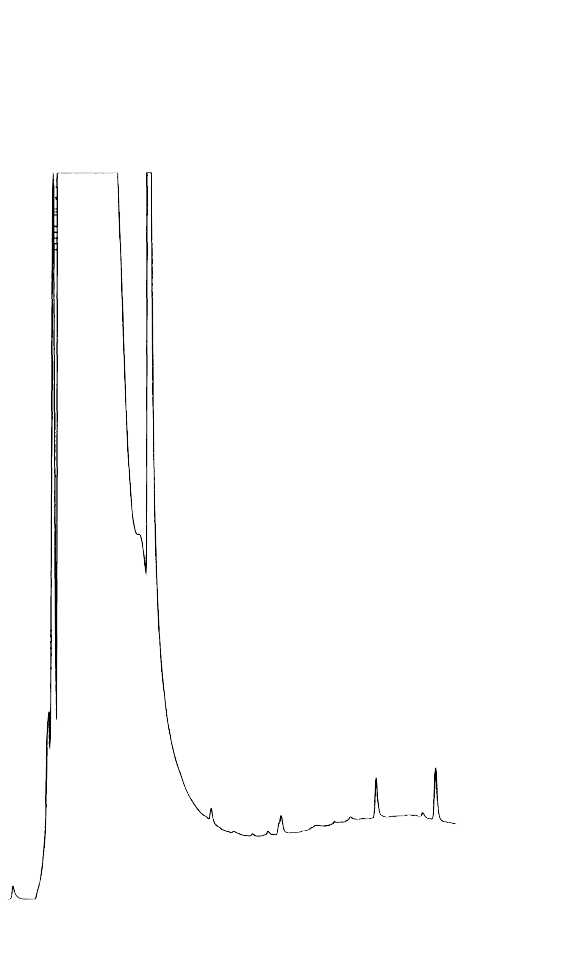

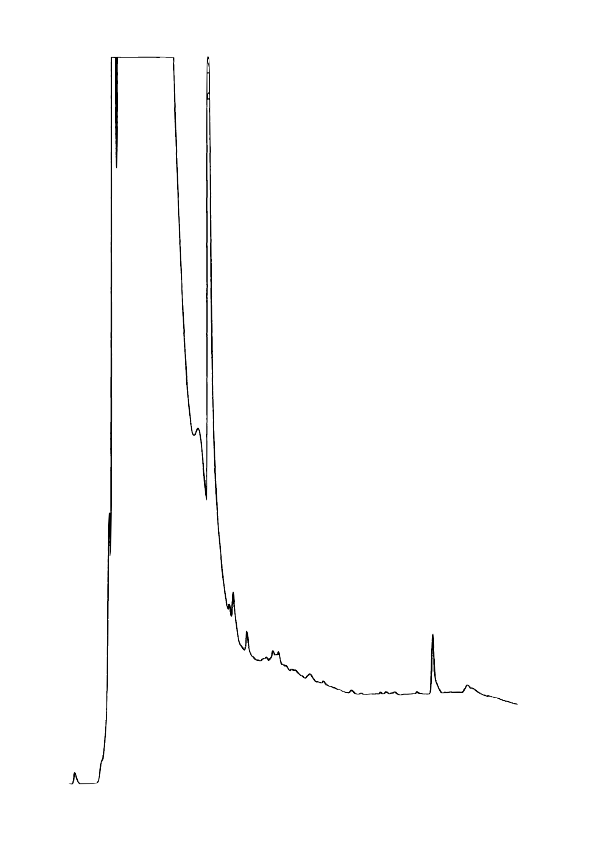


S30-1

S13-3


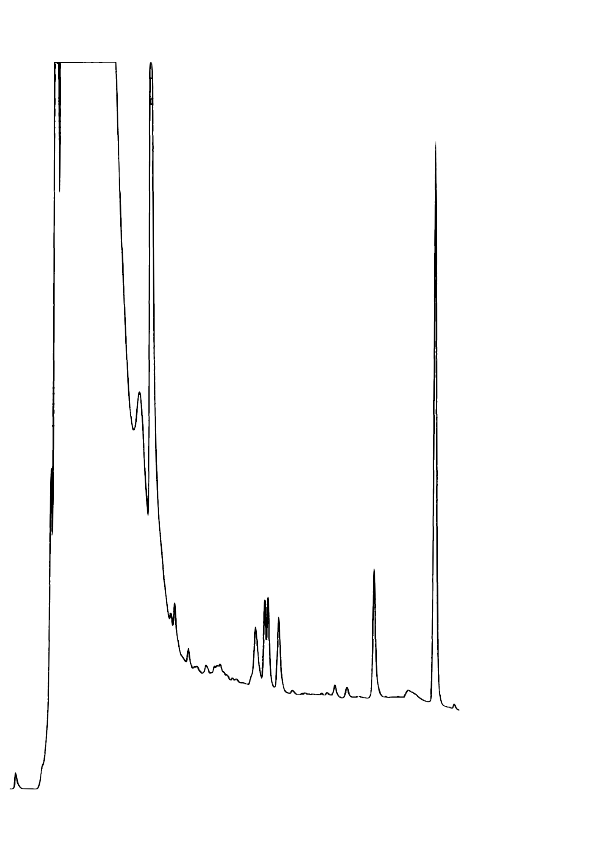


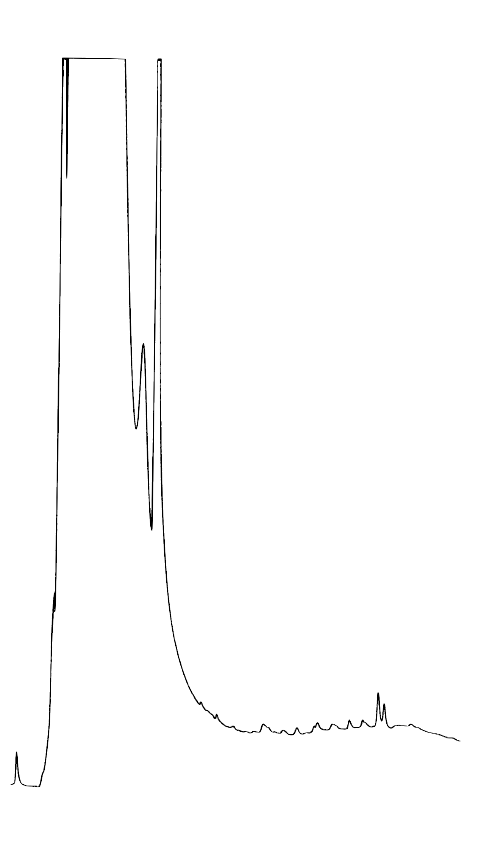

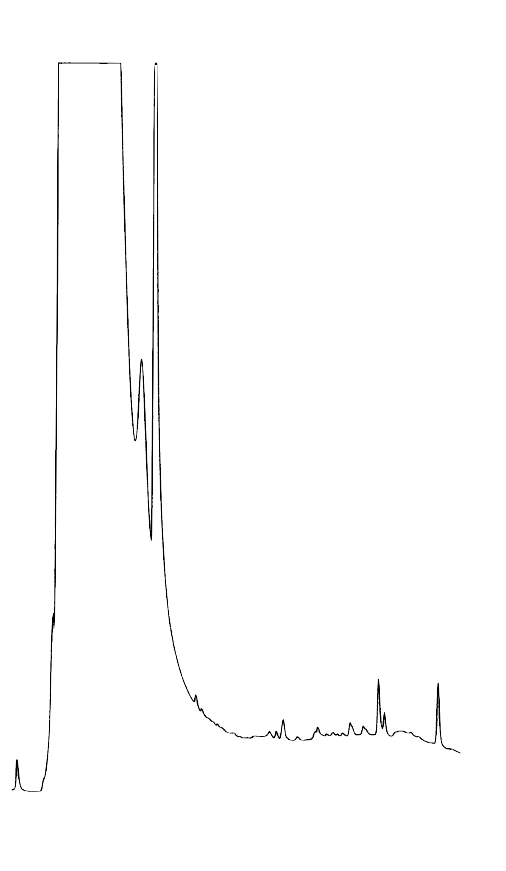

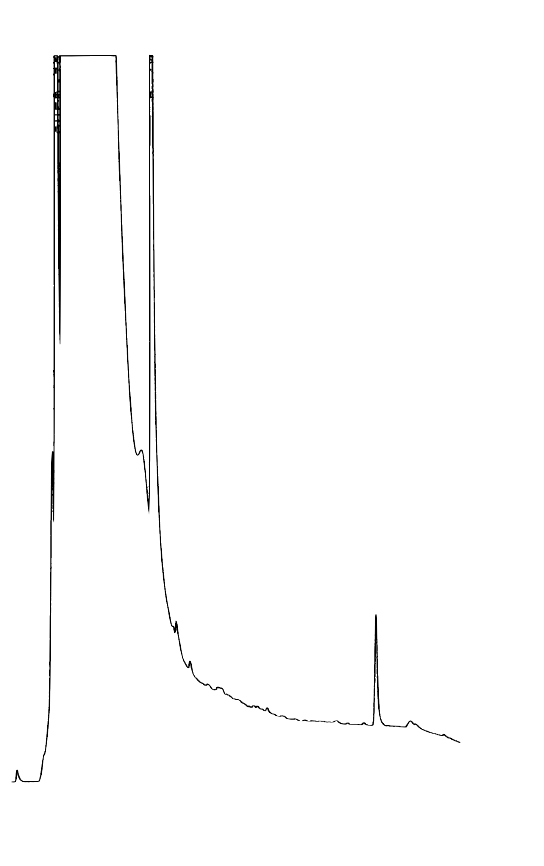


S37-1


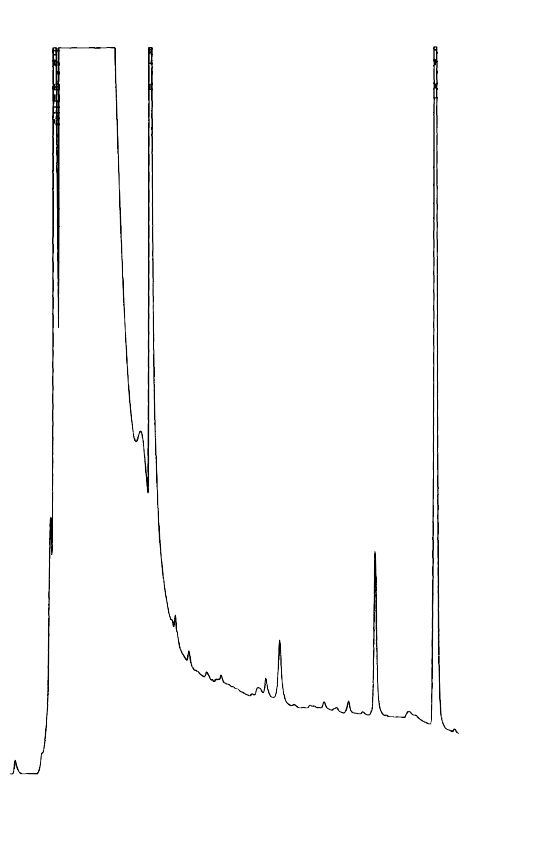


S46-1

S14-2


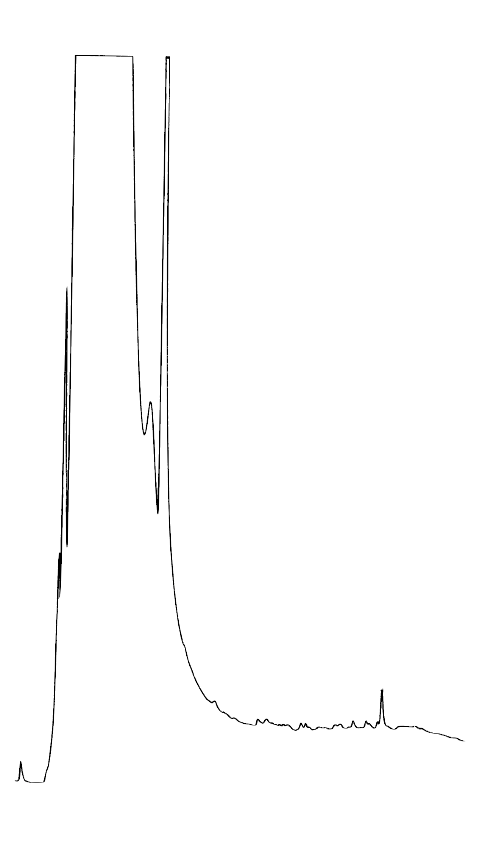

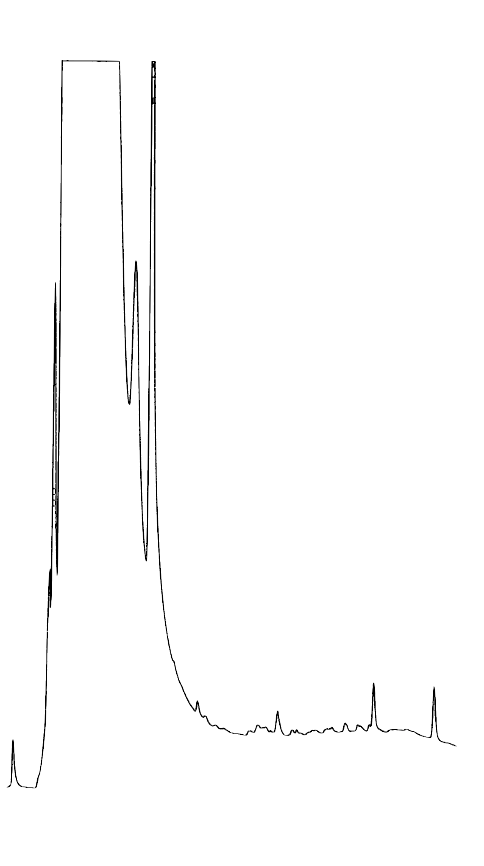

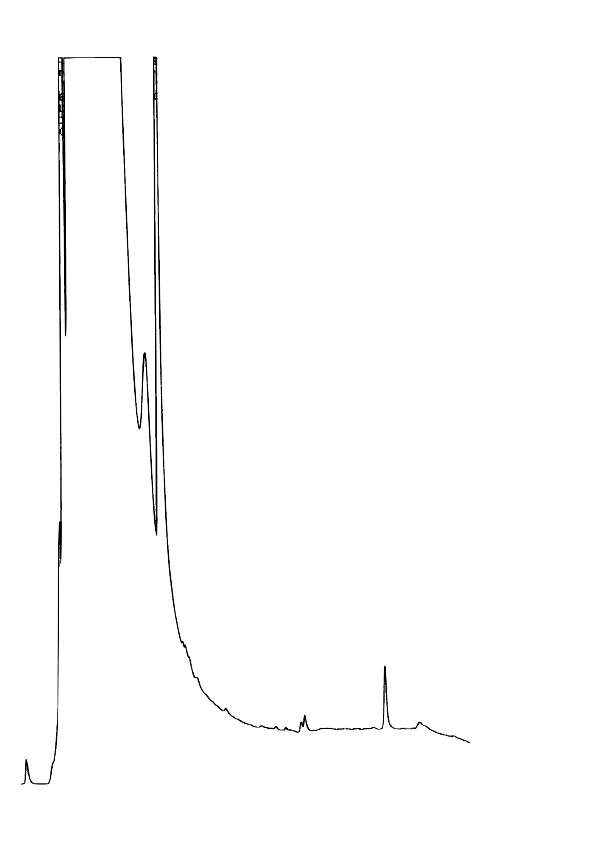


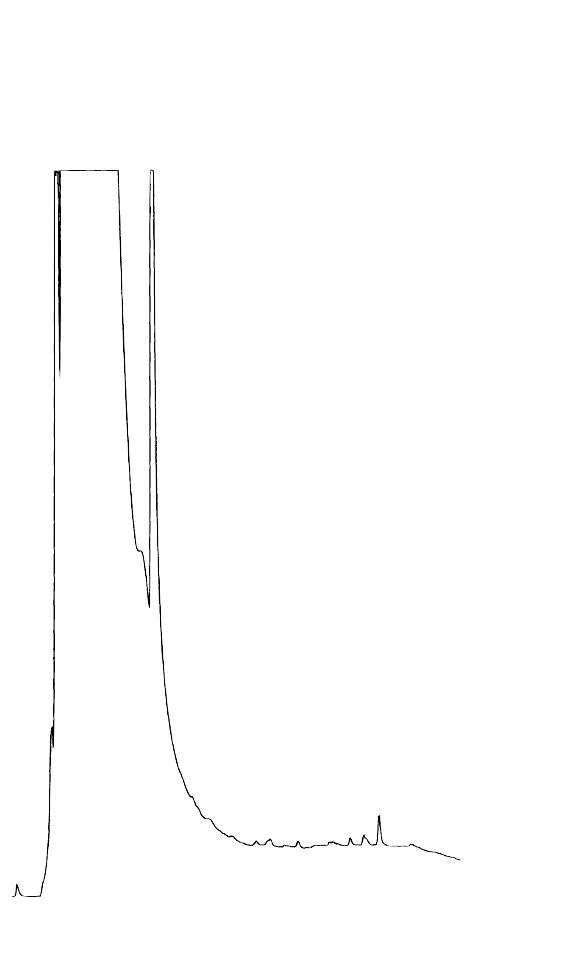

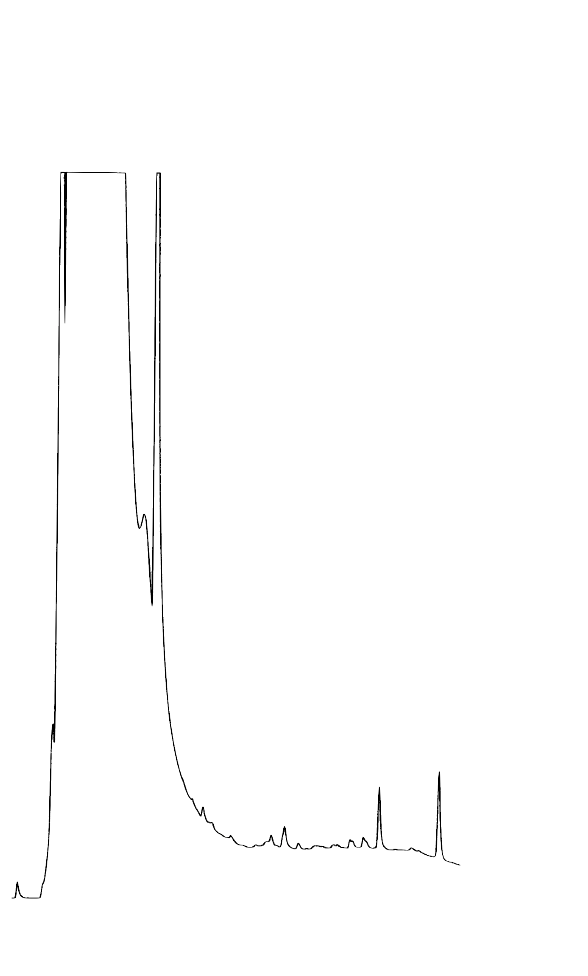


S48-1

S16-1


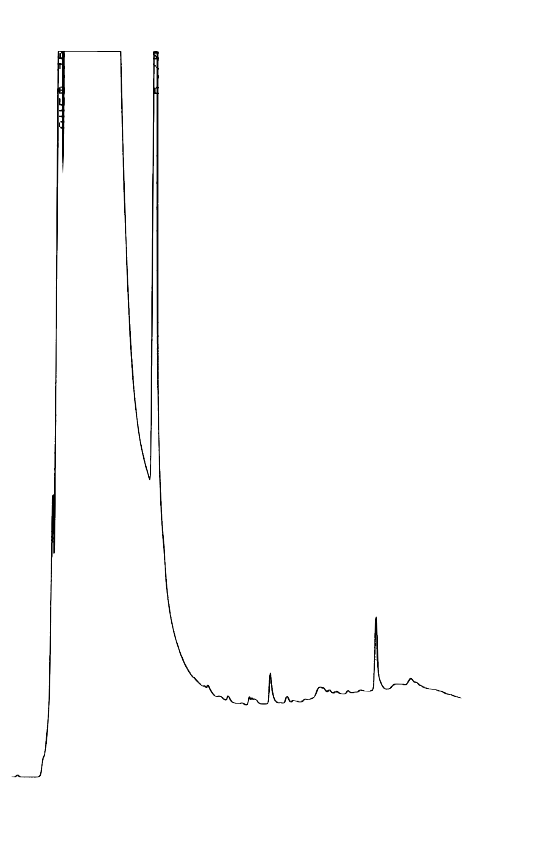


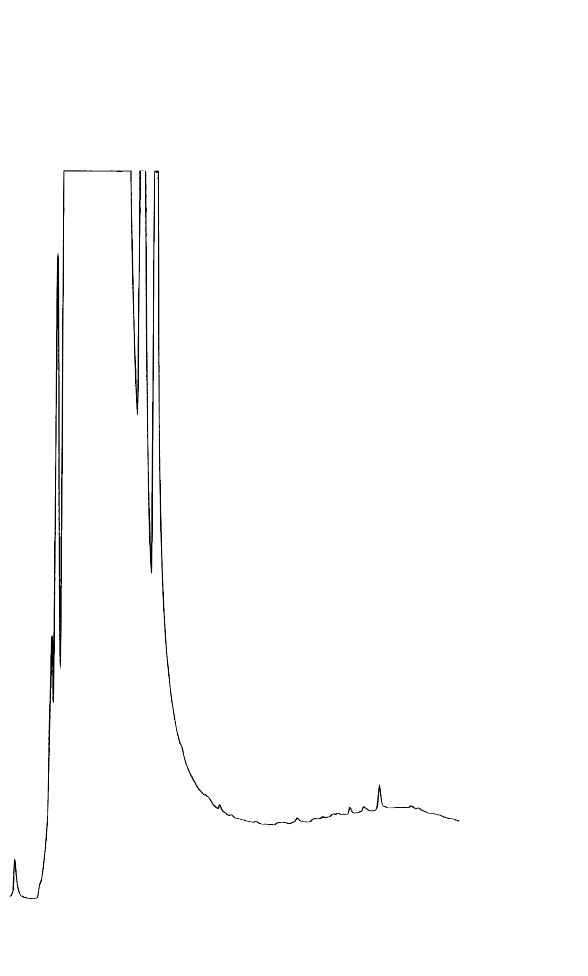

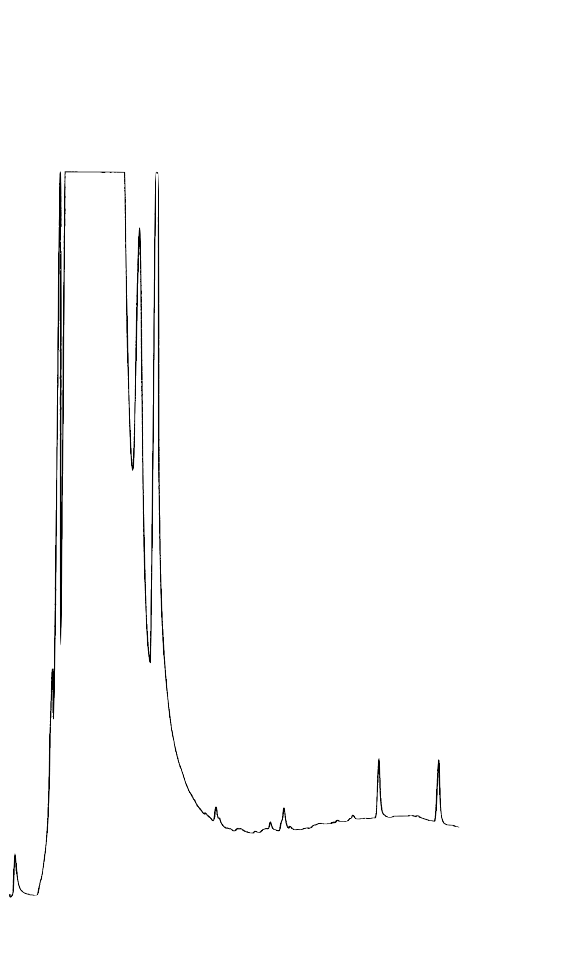


S53-1

S17-1


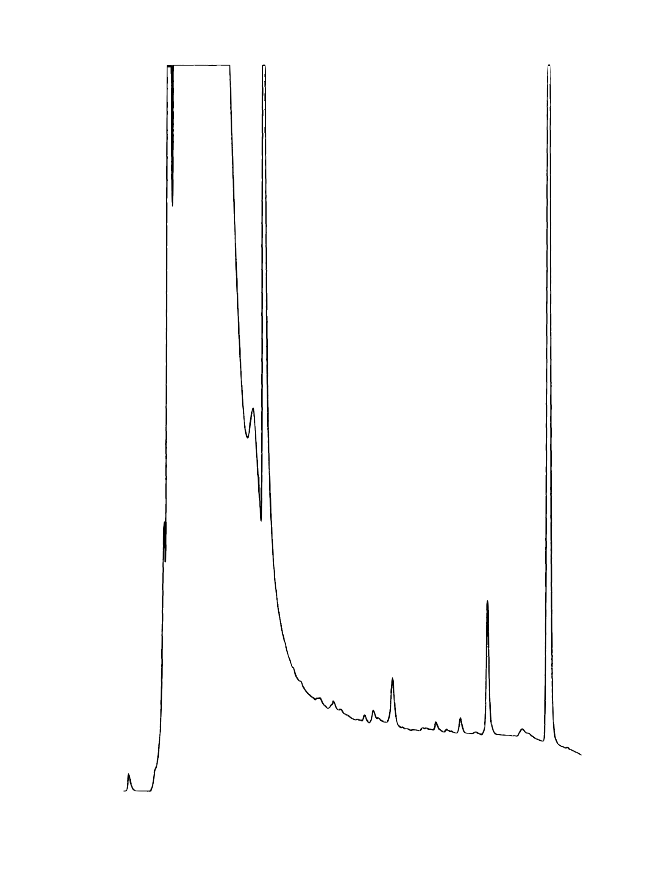


S53-2

S17-2


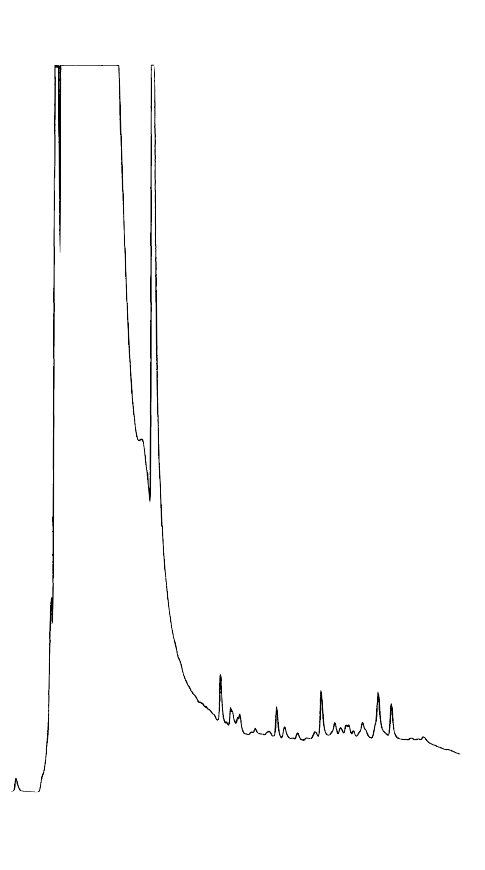

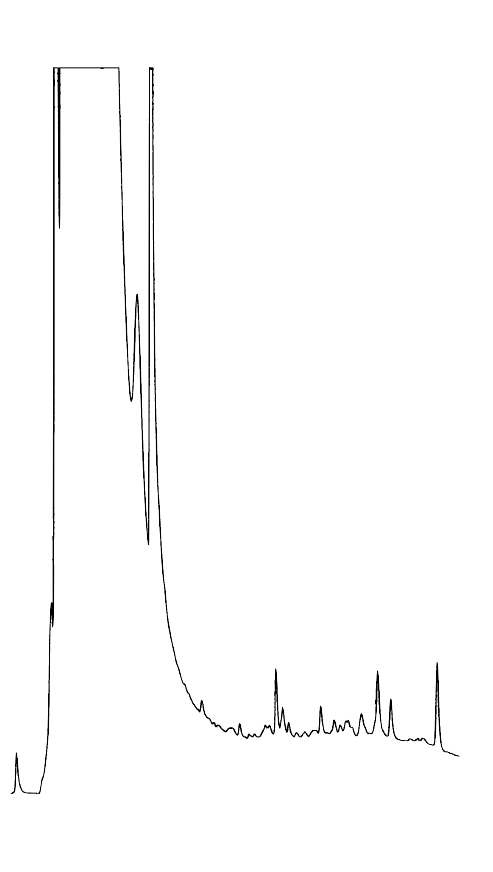

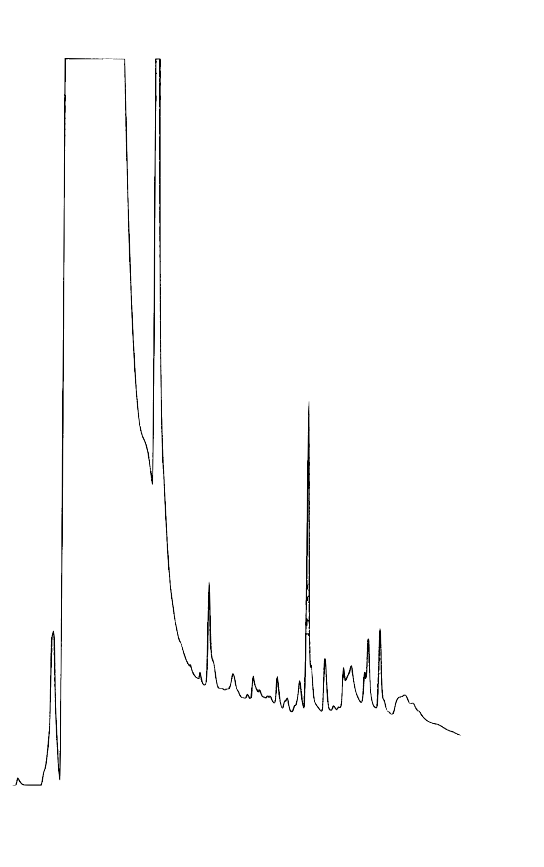


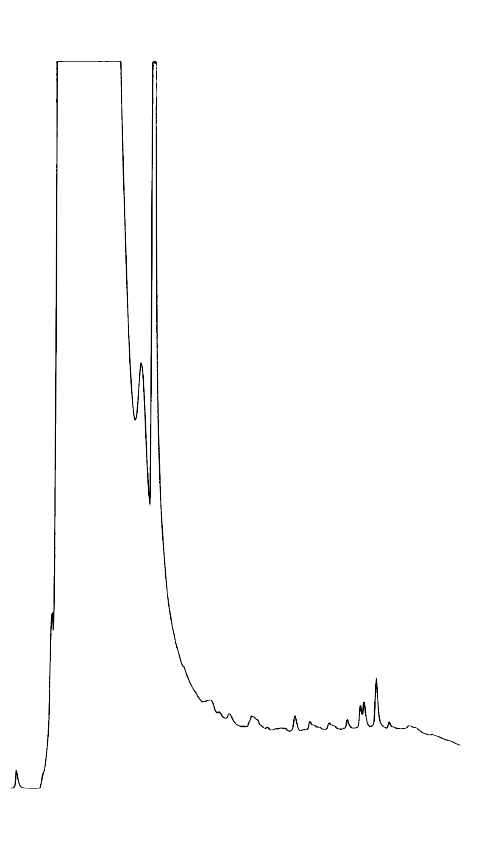


S54-1

S22-1


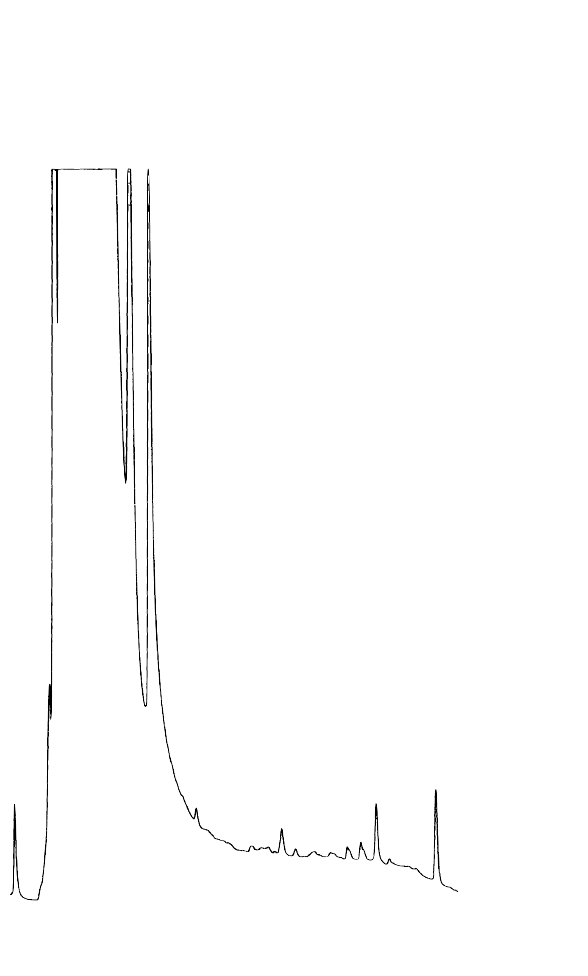

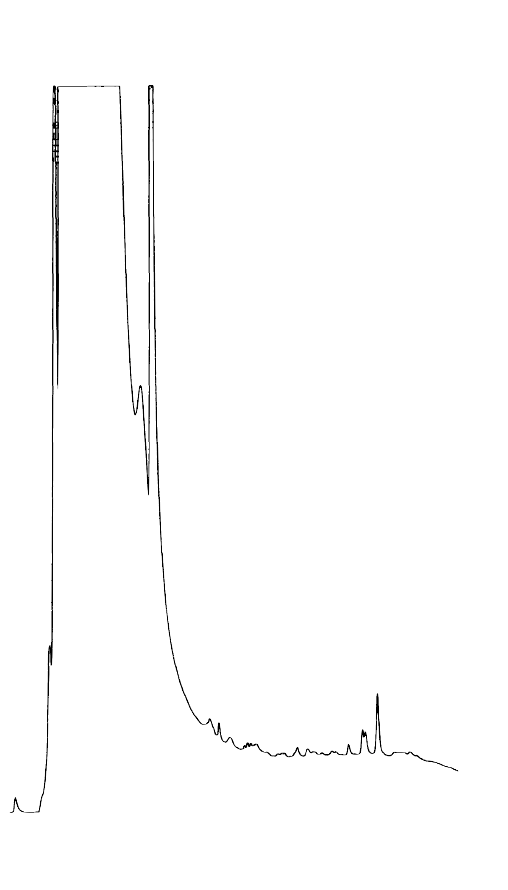


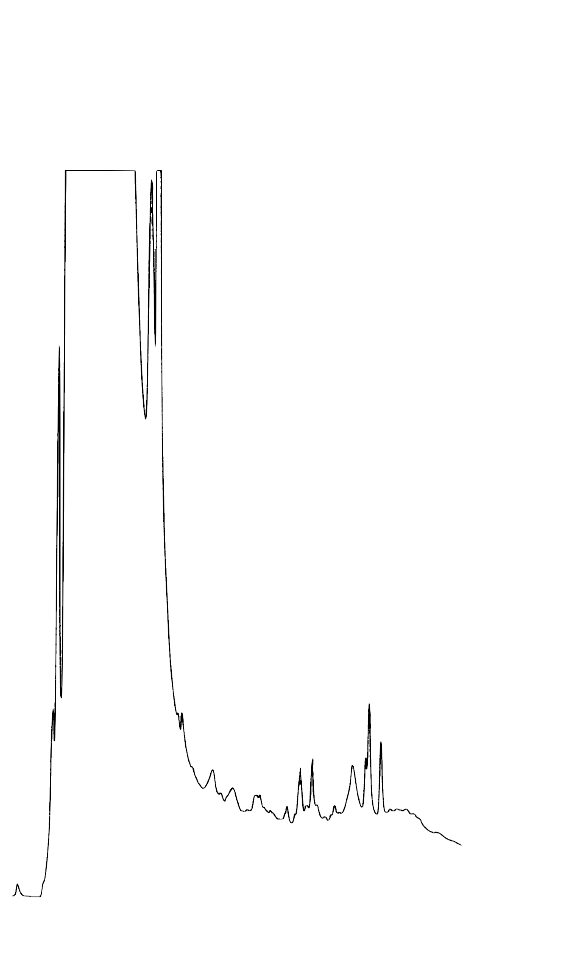


S54-2


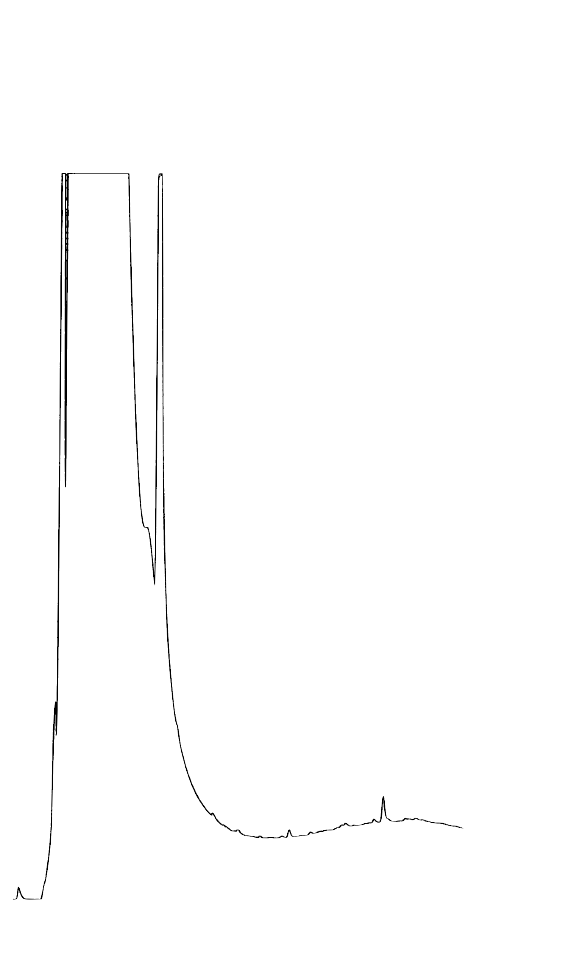

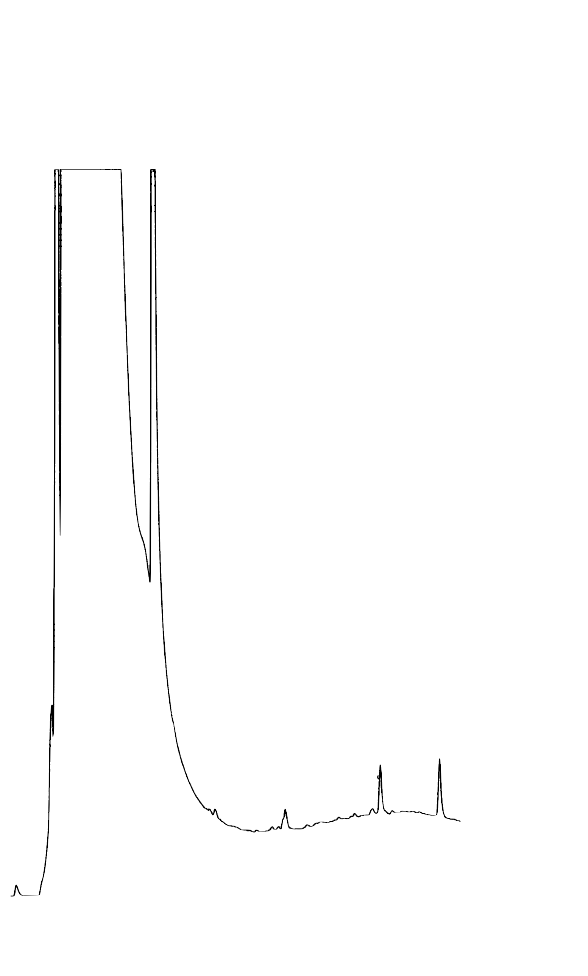


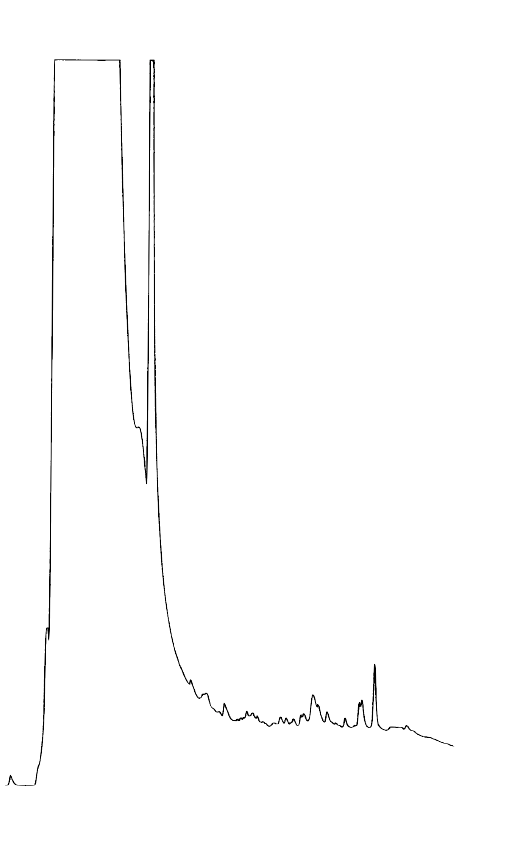

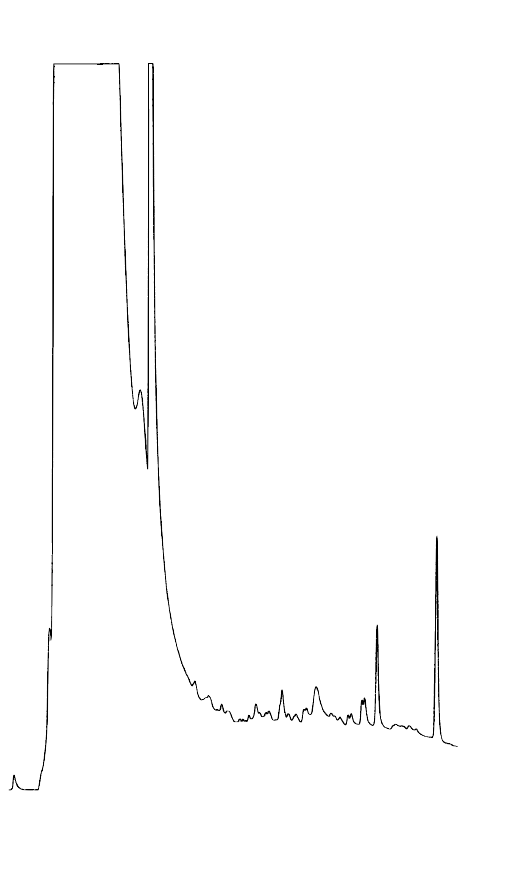

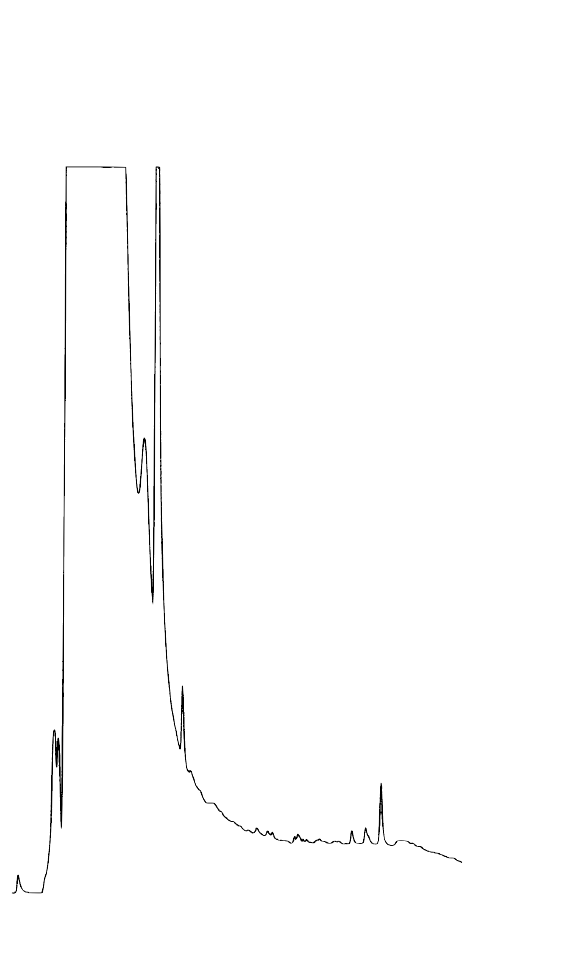


S57-1

S58-1

S27-1

8

10

15

20

(min)

8

10

15

20

(min)

8

10

15

20

(min)

**Figure S1**. Continued.


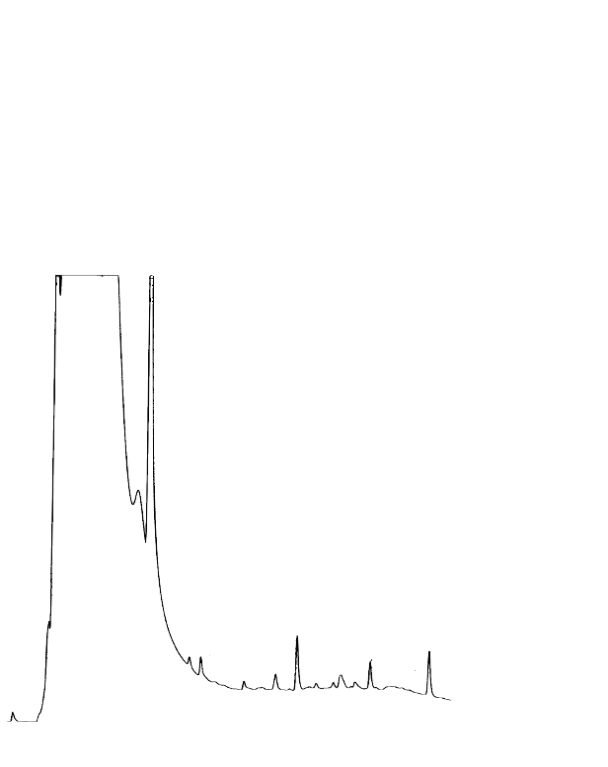

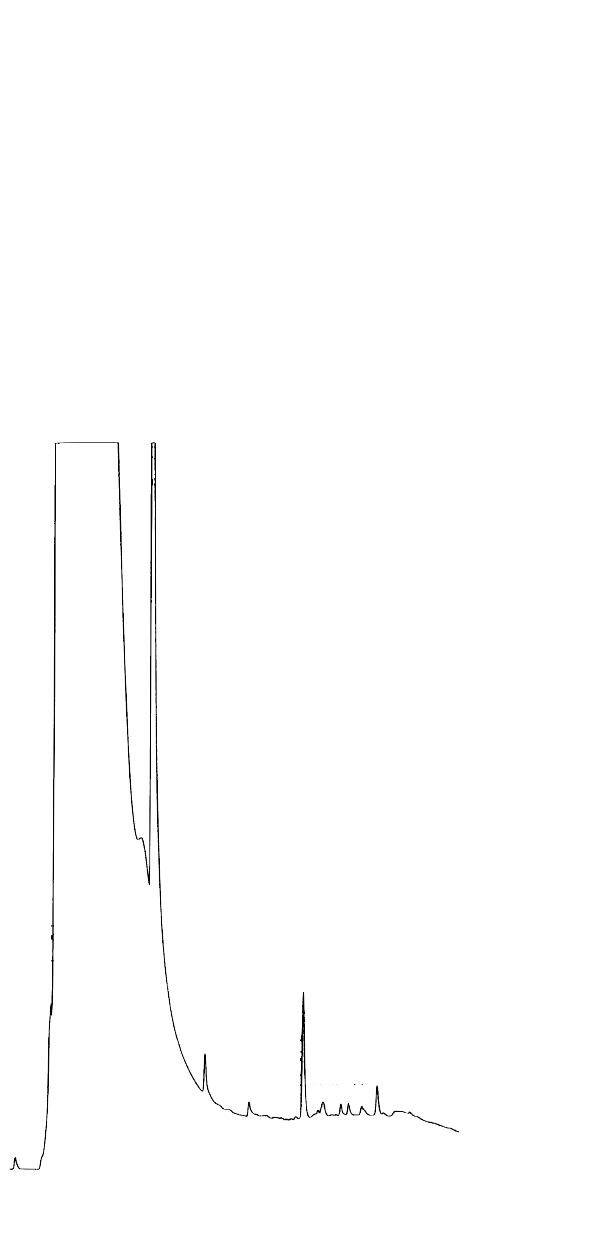


(b)

(a)

S58-2


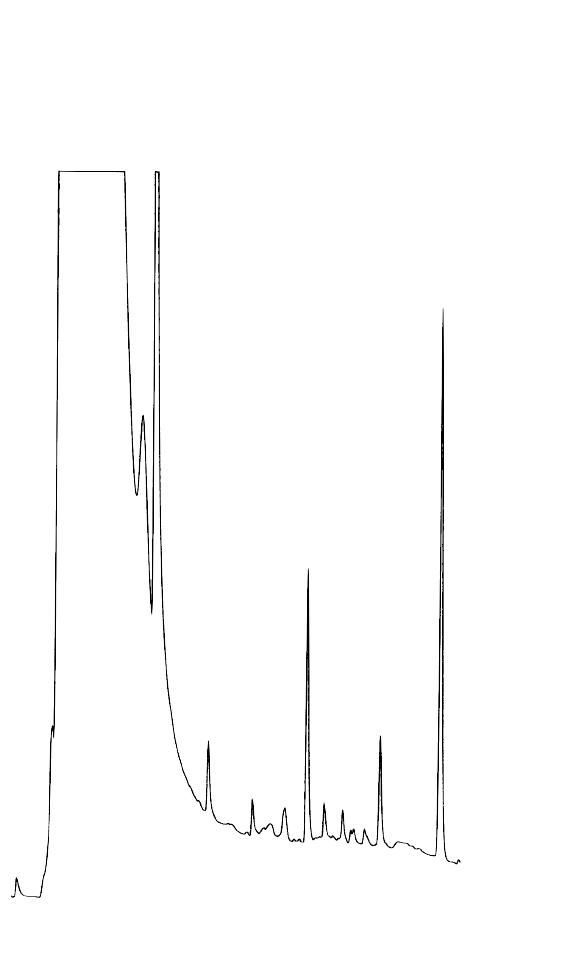


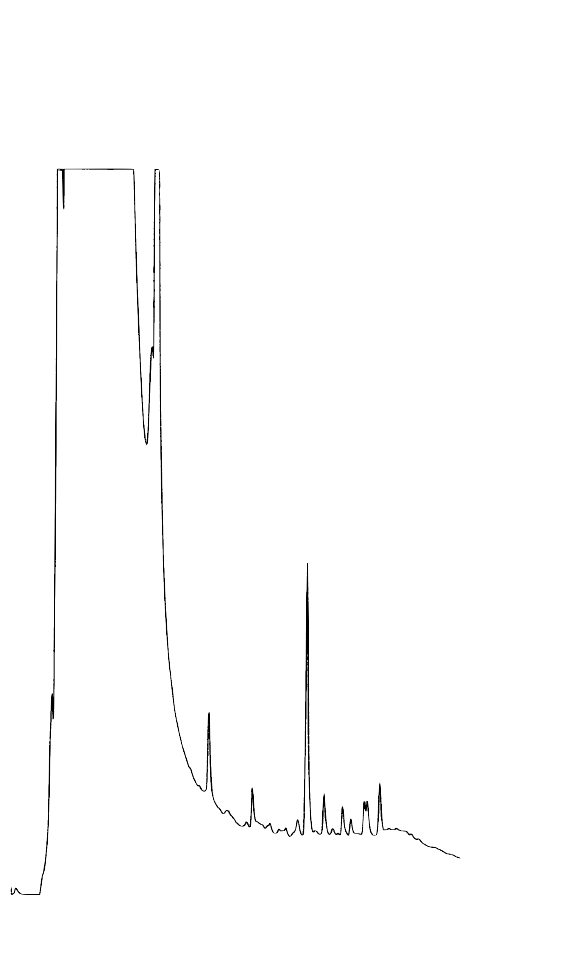


S100-1


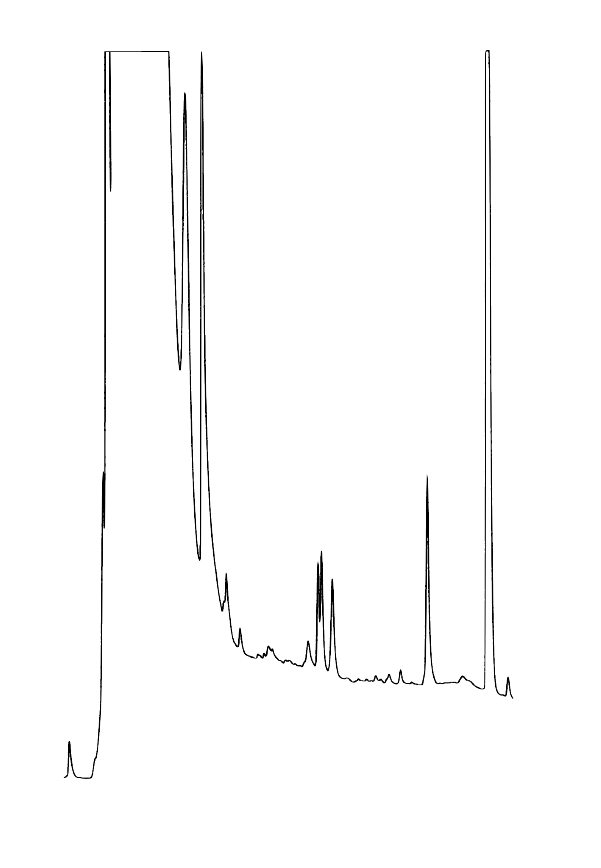


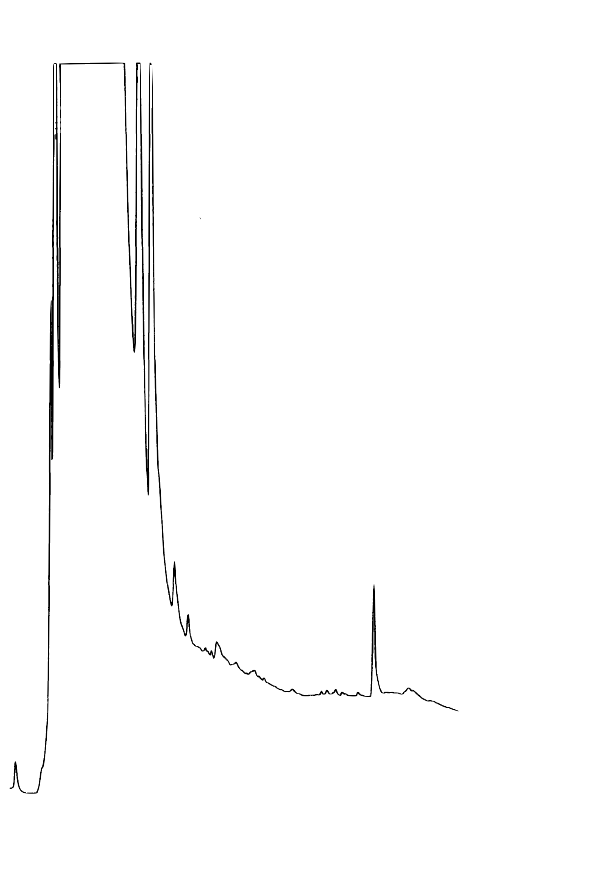


S113-1


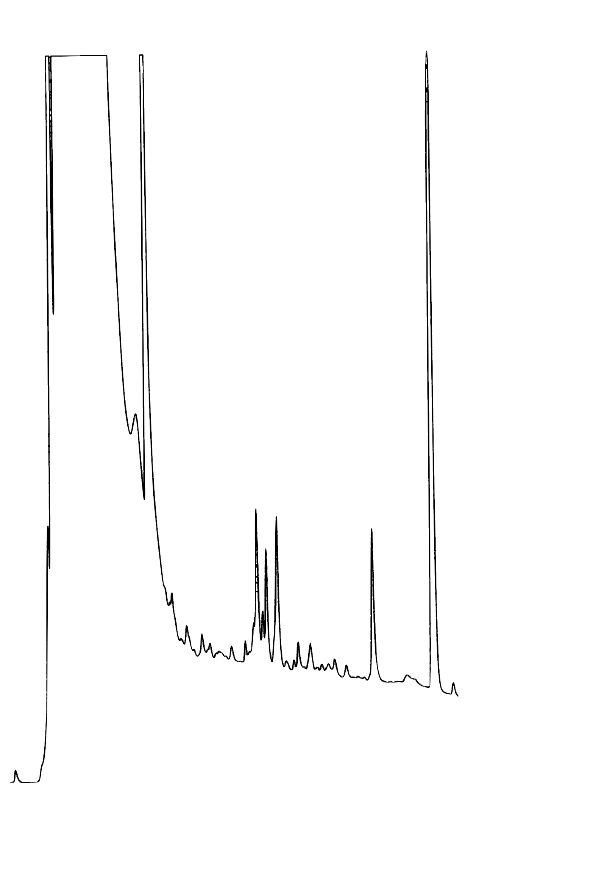

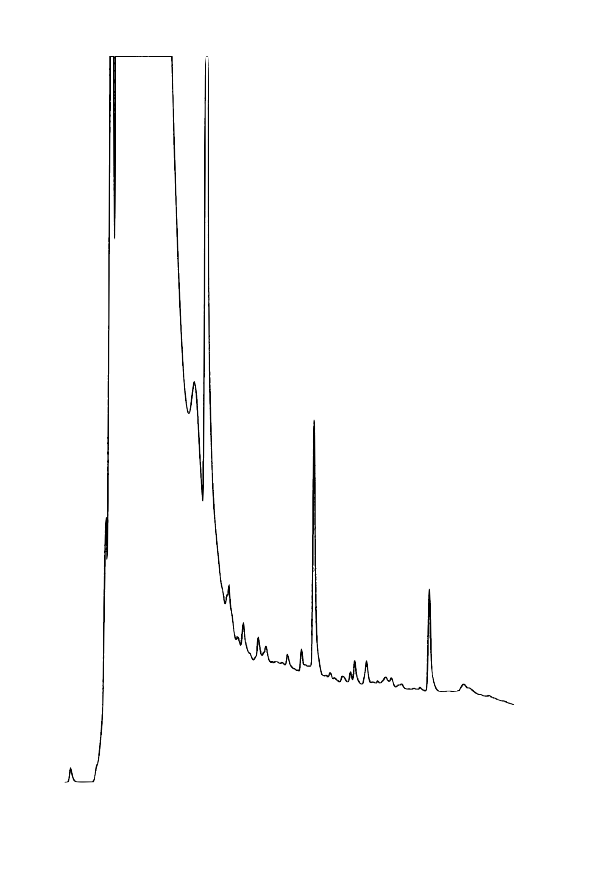


K91-1


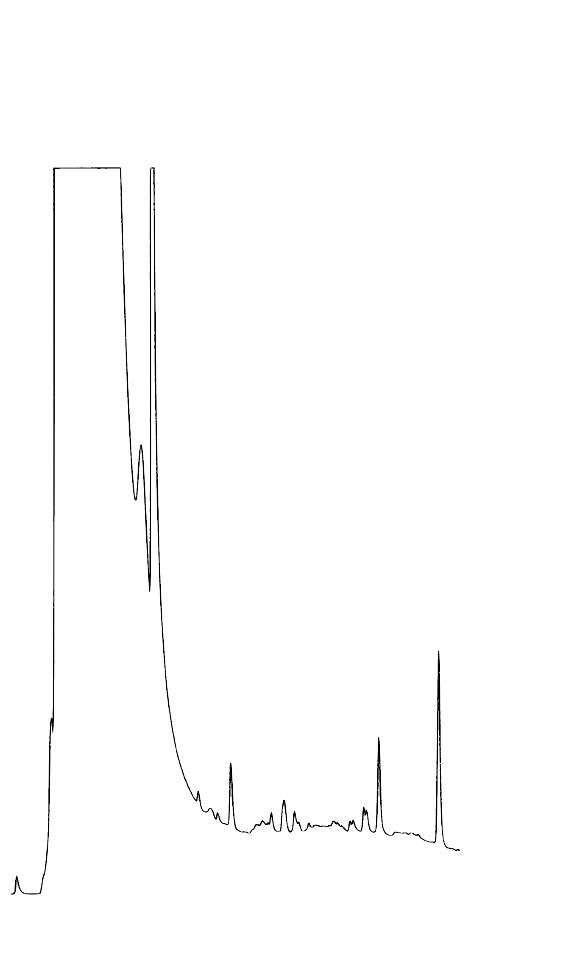

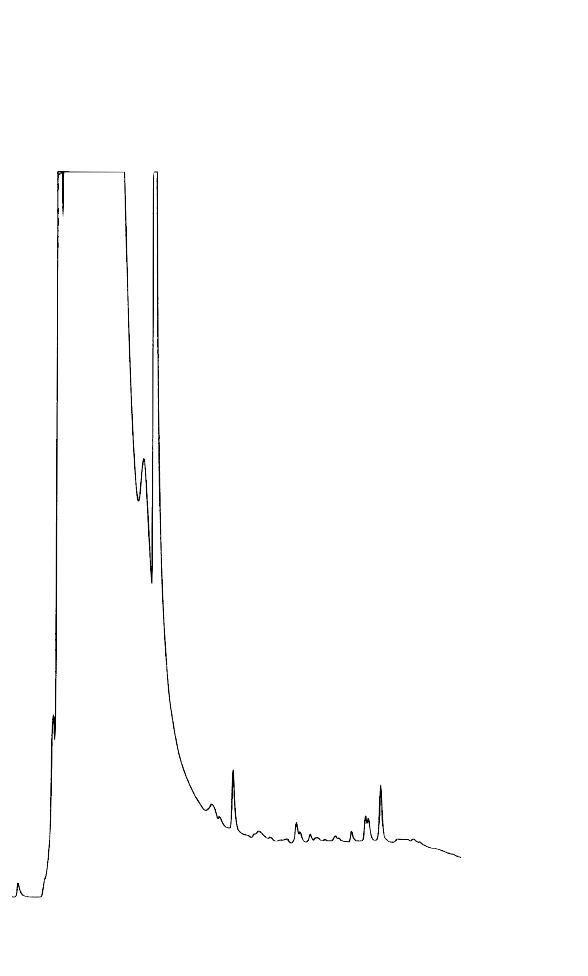


8

10

15

20

(min)

K95-1

8

10

15

20

(min)

**Figure S1**. Continued.
